# Guiding future research on psychological interventions in people with COVID-19 and post COVID syndrome and comorbid emotional disorders based on a systematic review

**DOI:** 10.3389/fpubh.2023.1305463

**Published:** 2024-01-11

**Authors:** Verónica Martínez-Borba, Laura Martínez-García, Óscar Peris-Baquero, Jorge Osma, Esther del Corral-Beamonte

**Affiliations:** ^1^Institute for Health Research Aragón (IIS Aragón), Zaragoza, Spain; ^2^Department of Psychology and Sociology, Universidad de Zaragoza, Zaragoza, Spain; ^3^Villanova Royo Hospital, Zaragoza, Spain

**Keywords:** COVID-19 patients, long COVID-19 conditions, psychological interventions, systematic review, emotional disorders

## Abstract

**Objective:**

The COVID-19 pandemic has been emotionally challenging for the entire population and especially for people who contracted the illness. This systematic review summarizes psychological interventions implemented in COVID-19 and long COVID-19 patients who presented comorbid emotional disorders.

**Methods and measures:**

3,839 articles were identified in 6 databases and 43 of them were included in this work. Two independent researchers selected the articles and assessed their quality.

**Results:**

2,359 adults were included in this review. Severity of COVID-19 symptoms ranged from asymptomatic to hospitalized patients; only 3 studies included long COVID-19 populations. Similar number of randomized controlled studies (*n* = 15) and case studies (*n* = 14) were found. Emotional disorders were anxiety and/or depressive symptoms (*n* = 39) and the psychological intervention most represented had a cognitive behavioral approach (*n* = 10). Length of psychological programs ranged from 1–5 sessions (*n* = 6) to 16 appointments (*n* = 2). Some programs were distributed on a daily (*n* = 4) or weekly basis (*n* = 2), but other proposed several sessions a week (*n* = 4). Short (5–10 min, *n* = 4) and long sessions (60–90 min, *n* = 3) are proposed. Most interventions were supported by the use of technologies (*n* = 18). Important risk of bias was present in several studies.

**Conclusion:**

Promising results in the reduction of depressive, anxiety and related disorders have been found. However, important limitations in current psychological interventions were detected (i.e., duration, format, length, and efficacy of interventions were not consistently established across investigations). The results derived from our work may help to understand clinical practices in the context of pandemics and could guide future efforts to manage emotional suffering in COVID-19 patients. A stepped model of care could help to determine the dosage, length and format of delivery for each patient.

**Systematic review registration**: PROSPERO 2022 CRD42022367227. Available from: https://www.crd.york.ac.uk/prospero/display_record.php?ID=CRD42022367227

## 1 Introduction

The coronavirus disease 2019 (COVID-19), caused by severe acute respiratory syndrome coronavirus 2 (SARSCoV-2), is considered one of the largest pandemics in world history and was declared a Public Health Emergency of International Concern by the World Health Organization (WHO) on January 30th, 2020 (1). COVID-19 symptoms range from asymptomatic or mild to severe (2), being fever, coughing, fatigue, and dyspnea the most prevalent physical symptoms of diagnosed patients (3). Thus, COVID-19 has caused high morbidity and mortality worldwide. As of 20 June 2023, it has affected more than 768 million people and caused nearly 7 million deaths (4). Special attention should be paid to COVID-19 patients who survive the pandemics but do not recover their initial state of health and report persistent and/or new physical symptoms 3 months after the initial infection, which has been referred by the WHO as post COVID-19 syndrome (5). Thus, more specifically, within this condition the most frequent reported symptoms have been brain fog, dizziness, loss of attention, confusion, chest pain, tachycardia, diarrhea, vomiting, general fatigue, dyspnea, and cough, among others (6).

The impact of the pandemic was observed not only in morbidity and mortality numbers; the pandemic situation and the measures taken during its duration, such as lockdown or reduction of social contact, have had a significant emotional impact on the entire population (7–11). It seems that it was a hard situation for millions of people, with a higher prevalence of psychological symptoms among those who suffered from the disease (12). One study found that patients who were quarantined due to COVID-19 infection showed psychological symptoms such as anxiety and depression symptomatology, lack of self-control and low levels of well-being and vitality (13). From all COVID-19 patients, a high proportion of mental health problems were observed in long or post COVID-19 populations, which presented high rates of persistent psychological distress (36%), anxiety disorders (22%), depression (21%), post-traumatic stress disorder (20%), and sleep disorders (35%) (12).

As we can see, there is a great variety and prevalence of physical and psychological symptoms related both directly to the COVID-19 infection and to the development of post COVID-19 syndrome after the infection. Thus, it has been claimed there is a need for multidisciplinary interventions to address the physical and psychological symptoms associated with COVID-19 (14). From a physical perspective, we found different systematic reviews and meta-analyses on the efficacy of antiviral treatments for the reduction of mortality and risk of hospitalization of patients infected with COVID-19 (15). Probiotics, prebiotics, synbiotics, and postbiotics for the modulation of the microbiota have been used in COVID-19 patients with the aim of reducing the severity and duration of symptoms such as dyspnea, olfactory dysfunction, nausea, vomiting, and gastrointestinal problems (16). In the case of the Post-COVID condition, specific rehabilitation programs have been developed with the input of multidisciplinary professionals (i.e., physiotherapists, occupational therapists, speech and language therapists, social workers, neuropsychiatrists, dieticians or nutritionists, among others) (17).

Similarly, from a psychological perspective, we found different systematic reviews in the field of psychological interventions for COVID-19 patients (18, 19). Promising results were found in the reduction of emotional suffering in COVID-19 patients, which suggest that psychological issues could be properly treated in the context of COVID-19 conditions. However, we noted some important limitations in these systematic reviews. First, some of them have summarized interventions focused mainly on COVID-19 patients which did not include long COVID conditions (18). Second, increased attention has been paid to severe cases (i.e., hospitalized patients) (19) or other non-COVID-19 populations (i.e., relatives, professionals, general populations) (20). Third, despite the well-known comorbidity between anxiety and depressive symptoms in COVID-19 patients, other systematic reviews addressed isolated depressive symptoms (21), or anxiety and related disorders (22).

With the aforementioned information in mind, to the best of our knowledge, this is the first systematic review which aims to explore and update the main characteristics of the psychological interventions delivered to patients with COVID-19 or long COVID-19 conditions and comorbid emotional disorders or symptoms. Results derived from this work may help to guide future clinical and research efforts conducted on the management of these patients.

## 2 Materials and methods

### 2.1 Eligibility criteria

According to the main objective of this systematic review, inclusion criteria to select the scientific articles were: (a) a psychological intervention was provided; (b) patients presented with COVID-19 or long-COVID-19 conditions; (c) changes in psychological outcomes were reported; (d) patients presented with emotional disorders or symptoms; (e) COVID-19 patients were the main participants; (f) the full text of the articles was written in English or Spanish. Similarly, pre-specified exclusion criteria included: (a) psychological program was not provided; (b) patients did not present with any form of COVID-19 condition; (c) psychological outcomes were not reported; (d) patients presented with severe mental disorders (i.e., psychotic disorders); (e) intervention was focused exclusively on relatives, professionals or general population. Other exclusion criteria had to do with manuscript type and the design of the study. This way, records were excluded for synthesis if they were not scientific articles (i.e., book chapters or conference papers) or they were protocol studies or trial registrations. Additionally, papers were excluded if they were systematic reviews/meta-analysis or if they do not provide efficacy data (i.e., theoretical description of interventions without efficacy results).

### 2.2 Search strategy

This systematic review has been conducted in accordance with the Preferred Reporting Items for Systematic Reviews and Meta-analyses [PRISMA, (23). See Supplementary materials A, B]. The literature search was carried out in specialized databases in the field of mental health and health conditions. Specifically, literature searches were performed in WOS (Web of Science), Scopus, PubMed, PsycINFO, Cochrane and CINAHL. The following word combinations and Boolean operator were entered in the databases: “COVID-19 conditions” AND/OR “psychological interventions” AND/OR “psychological issues” (see a detailed description on Supplementary material C). No language or data restrictions were applied in the searches, which were conducted by two independent researchers (*VM-B and LM-G*) on June 14, 2023. In addition to the database search, reference lists of different systematic reviews and meta-analyses were also examined to identify the possible inclusion of articles that were not initially found in the databases.

Regarding the management of the results, we used the Mendeley platform (24), for the automatic elimination of duplicate results, and the Rayyan platform (25), for the subsequent review of the inclusion and exclusion criteria of the articles. For this purpose, two independent researchers (*VM-B and LM-G*) carried out the review of articles in two phases. The first consisted in checking the titles and abstracts of the articles to verify if they met the inclusion criteria. The second phase consisted in a complete reading of the articles selected in the first phase by the researchers to ensure that they, in fact, met the inclusion criteria. A third expert researcher (*JO*) was consulted when there were doubts about whether a specific article should be included or excluded.

### 2.3 Data extraction

To conduct the extraction of the information, a pre-specified list of outcomes was used by two independent authors (*VM-B and LM-G*). If there were any disagreements, a third author was consulted (*JO*). All studies included in the review were eligible for data extraction and synthesis. The pre-specified list was elaborated following the Cochrane recommendations (26). Additionally, in this systematic review we have include interpretation of results to identify whether improvements on psychological measures indicate a total recovery of symptoms (e.g., participants’ scores at post-intervention were below the clinical cut-off established for each questionnaire) or a partial recovery of symptoms (e.g., a decrease in the scores of psychological issues was observed but scores were above the clinical cut-off after the intervention). In this regard, some missing or unclear data was found in the extraction of the information. In some cases, the studies did not report the clinical cut-off that was used for a given questionnaire. To avoid reporting bias, we have checked if authors provided the reference of the questionnaire used in the study. If the reference of the questionnaire was reported, we used it as a cut-off. On the other hand, if the reference of the questionnaire was not mentioned, we used the original version of the questionnaire to determine whether a partial or a total recovery was obtained in this study.

In relation to presentation of results, we have reported the effect measure indicated in the study (i.e., means comparisons, effect sizes, reliable change index). Data presentation is supported by tables and was based on the information directly obtained from the article without converting the data. We have presented the information of such studies according to their design (case studies outcomes are reported in Table 1 while results from intervention studies with and without control group are presented in Table 2). No additional statistical analyses were calculated. Thus, meta-analysis, sub-group analysis and meta-regression and sensitivity analyses were not conducted. To avoid duplication and to reduce possible bias, authors pre-registered the review protocol in PROSPERO (CRD4202236722) on October 19, 2022.

**Table 1:** Extraction of data for case studies (*N* = 14).

Author*, year, location	Sample	Medical history	COVID-19 characteristics	ED	Psychological intervention	Outcomes	Results	Total / partial recovery
Alkhamees, 2021 (27) Saudi Arabia	*N* = 1. Male 62 years old. Retired	No remarkable medical history. No familial antecedents.	COVID-19 patient (No date reported).	Obsessive-Compulsive Disorder.	Escitalopram and Cognitive Behavioral Therapy (CBT). Dropped out of CBT after the 2nd visit.	No formal assessment was reported.	Self-reported reduction in intrusive thoughts (60% reduction on a scale from 0 to 100). He reported that most of these thoughts had disappeared. He resumed his routine and daily activities.	No cut-off reported, neither size effect nor significance of change was calculated.
Bogucki, 2022 (28) United States	*N* = 1. Female 30 years old. Partnered. College degree. Employed within the health care sector.	No psychiatric antecedents.	Diagnosed with COVID-19 in April 2020. Re-diagnosed with COVID-19 in mid-June 2020. Diagnosed with Post-COVID-19 syndrome in October 2020.	PTSD.	Cognitive Processing therapy (CPT): identifying, evaluating and restructuring cognitive distortions related to traumatic events. One session per week, 12 sessions.	PCL-C: 30–35 = general population; 36–44 = specialized medical clinics; 45–50 = mental health clinics. PHQ-9. GAD-7.	Reduction in PCL-C (session 0 = 53 points – session 12 = 21 points; 60% reduction). PHQ-9 (score = 0) and GAD-7 (score = 5) remained under the clinical cut-off point at session 12.	According to the PCL-C cut-off used in this study, a total recovery in PTSD was found. According to the cut-off of the original version of the PHQ-9, a total recovery on depression was observed. According to cut-off of the original version of the GAD-7, a partial recovery on anxiety symptoms was found.
Callus, 2022 (29) Italy	*N* = 1, male, 31 years old.	In December 2019 psychotherapeutic care.	Diagnosed with COVID-19 in March 2020. Hospitalized.	Anxiety and panic.	22 sessions over 2 months: relaxation and breathing. Calls, videocalls, WhatsApp.	Online questionnaire: GAD PHQ ISI	In the final assessment no psychological problems were reported.	No cut-off reported, neither size effect nor significance of change was calculated.
Chen, 2020 (30) China	*N* = 1. Female 49 years old	Not reported.	Diagnosed January 22, 2020 Hospitalized.	Major Depressive Disorders	Psychotherapy. Pharmacotherapy (escitalopram, lorazepam).	DSM-5	Psychological symptoms improved and remitted.	No cut-off reported, neither size effect nor significance of change was calculated.
Dinapoli, 2022 (31) Italy	*N* = 12 (9 male; 3 female). 25–63 years old (mean = 52.3)	Not reported.	COVID-19 survivors (October 2020–February 2022)	PTSD.	Eye Movement Desensitization and Reprocessing Therapy (EMDR), 8–16 weekly treatment sessions. 10 patients were receiving pharmacotherapy.	Event Scale-Revised (IES-R); Adverse Childhood Experiences (ACE); Dissociative experiences Scale (DES-II; cut-off 30 = dissociative symptoms; 40 = PTSD)	A significant decrease was found in the IES (*p* = 0.005) ad DES (*p* = 0.032)	According to the cut-off reported in the study, participants had non-clinical symptoms of PTSD before the intervention (scores on DES below 30 points).
Edet, 2022 (32) Nigeria	*N* = 1. Male 27 years old. Unmarried. Tailor.	No remarkable medical and psychological history. No familial antecedents.	Diagnosed and recovered from COVID-19 (No date reported). Admitted into the ward due to psychological manifestations.	Major Depressive disorder with severe anxious distress. Severe suicidal ideation.	Supportive psychotherapy and pharmacotherapy (amitriptyline).	DSM-5	Symptoms resolved in six weeks. He resumed work nine weeks after discharge. Clinical symptoms were not present at 12 weeks after discharge.	No cut-off reported, neither size effect nor significance of change was calculated.
Hu, 2020 (33) China	*N* = 1 Doctor, employed in a rural clinic.	No remarkable medical history.	Beginning of symptoms in January 2020. Hospitalization in an infectious disease hospital.	Severe anxiety and depression.	Interpersonal Psychotherapy (IPT): empathy, psychoeducation “sick role,” family communication. 3 sessions in one week. No pharmacotherapy.	GAD HAMA PHQ-9 HAMD-17	GAD-7 reduced from 20 to 5 points. HAMA decreased from 41 to 6 points PHQ-9 decreased from 21 to 2 points. HAMD-17 decreased from 23 to 2 points.	According to cut-off of the original version of the PHQ-9, a total recovery on depressive symptoms was observed. According to cut-off of the original version of the GAD-7 a partial recovery on anxiety symptoms was found.
Huang, 2020 (34) China	*N* = 1. Female 30 years old. Married, with a daughter and currently pregnant (35 weeks of gestation).	Not reported	Diagnosed with COVID-19 in February 2020. Hospitalization in the hospital	Depression and Anxiety.	Dialectical Behavioral Therapy (DBT): release intensive emotion, psycho-education, mindfulness, breathing, distress tolerance and interpersonal skills. 3 sessions. No pharmacotherapy.	HAMD-17. MADRS. HAMA.	HAMD-17 scores were reduced from 13 to 3 points. MADRS scores decreased from 19 to 2 points. HAMA scores decreased from 15 points to 1.	According to the HAMA cut-off used in this study, a total recovery on anxiety symptoms was found. According to the MADRS cut-off used in this study a total recovery on depressive symptoms was found.
Khawam, 2020 (35) United States	*N* = 1 Female, 62 years old, divorced, two children. Retired.	Lifelong generalized worrier. History of panic attacks.	Diagnosed with COVID-19 (No date reported). Hospitalized.	Generalized anxiety disorders and cute anxiety attacks.	Daily support psychotherapy (telephone). Pharmacotherapy (Lorazepam, gabapentin and melatonin).	Clinical interview.	Anxiety symptoms improved with her respiratory symptoms.	No cut-off reported, neither size effect nor significance of change was calculated.
Naskar, 2022 (36) India	*N* = 1 Female. 27 years old. Health-care worker.	Recurrent Depressive Disorders.	Diagnosed with COVID-19 (No date reported). Hospitalized in ward isolation.	Severe depression. Self-harm attempt.	Psychotherapy: twice daily sessions, video conferencing. Pharmacotherapy (Escitalopram and clonazepam).	No formal assessment was reported.	Suicidal ideation was reduced in frequency and intensity. She resumed work after 1 week of staying at home.	No cut-off reported, neither size effect nor significance of change was calculated.
Nuertey, 2022 (37) Ghana	*N* = 2. Case 1: 30 years old, male. Case 2: 43 years old, male.	No remarkable psychological history.	COVID-19 patients on isolation wards.	Depression and suicide attempt.	Case 1: 13 counseling sessions (psychoeducation and cognitive therapy approach). Case 2: 4 sessions: social support, positive recovery, relaxation.	Case 1: DASS Case 2: Subjective Units of Distress (SUB)	Case 1: a reduction in anxiety (pre = 21 – post = 9), depression (pre = 24 – post = 12) and stress (pre = 25 – post = 12) was observed. Case 2: subjective Units of Distress decreased from 9 to 3 points.	No cut-off reported, neither size effect nor significance of change was calculated.
Sadeghi, 2021 (38) Iran	*N* = 3, English students. 20, 21 and 24 years old	Not reported.	COVID-19 survivors (No date reported).	PTSD Depression	Emotion-Focused Therapy (EFT): interpersonal communication and individual emotions. No pharmacotherapy.	PCL > 50. BDI. 0–13 = minimal; 14–19 = mild; 20–30 = moderate, >30 = severe depressive symptoms.	Reduction in PCL: P1 (BL = 72.3 – FU = 39.6, RCI = 5.88–5.81), P2 (BL = 78.0 – FU =49.0 RCI = 5.32–5.15), P3 (BL = 66.3 – U = 38.3, RCI 5.02–4.97). Reduction in BDI: P1 (BL = 27.3 – FU = 11.3, RCI = 5.65–5.54), P2 (BL = 25.0 – FU = 10.0, RCI = 5.55–5.21), P3 (BL = 29 – FU = 11.0, RCI = 5.91–6.25).	According to the cut-off of the PCL reported in this study, a total recovery on post-traumatic symptoms was found. According to the cut-off of the BDI reported in this study a total recovery on depressive symptoms was found.
Situmorang, 2021 (39) Indonesia	*N* = 1. Female, 33 years old, widow.	Not reported.	Asymptomatic COVID-19. Cared for in her own home.	Anxiety, panic, depression, stress, insomnia, delusions of death.	Music therapy: participant is encouraged to sing a song she loves and to create new lyrics using this song. One session.	Not reported.	At the end of the session, she reported that anxiety, panic, depression, stress, insomnia and delusions of death had decreased to 5.	No cut-off reported, neither size effect nor significance of change was calculated.
Taube, 2023 (40) Latvia	*N* = 1. female, 72 years old	Not reported.	Severe infection in October 2021. Re-infection, less symptoms in March 2022. Persistent COVID-19 symptoms.	Depression and anxiety.	Virtual art, music, drama, dance, movement therapy, psychotherapy and occupational therapy. Pharmacotherapy (antidepressants).	Clinical Global Impressions Scale (CGI-S) PHQ-9	She continued with psychiatrist after hospital discharge. Her mood improved, she had more energy and coped with daily tasks. She did not experience anxiety.	No cut-off reported, neither size effect nor significance of change was calculated.

*We have included only the first authors’ name. CBT, Cognitive Behavioral Therapy; PCL-C, PTSD Checklist-Civilian Version; PHQ-9, Patient Health Questionnaire-9; GAD-7, Generalized Anxiety Disorder Scale-7; DSM-5, Diagnostic and Statistical Manual of Mental Disorders; HAMA, Hamilton Anxiety Scale; HAMD-17, Hamilton Depression Rating Scale-17; MADRS, Montgomery-Asberg Depression Rating Scale; PTSD, Post-traumatic Stress Disorder; RCI, Reliable change index; BL, baseline; FU, follow-up; ISI, Insomnia Severity Index; DASS, Depression Anxiety Stress Scales.

**Table 2:** Extraction of data for controlled and non-controlled studies (*N* = 29).

Author*, year, location	Study design	Sample	COVID-19 characteristics	ED	Psychological intervention	Outcomes (instrument, cut-off)	Results	Total / partial recovery
Biagianti, 2023 (41) Italy	Pre-post without control group. Recruitment: up to June 30th, 2022	*N* = 102 (73 patients, 29 relatives), 64% female, age = 49.1 (*SD* = 16.0).	COVID-19 patients and their relatives.	Anxiety, depression	Telephone screening. 8 online sessions (zoom): emotional validation, interpersonal resources, regulation techniques and grief.	GAD (5 = mild; 10 = moderate; 15 = severe anxiety. Cut-off = 10) PHQ (5 = mild, 10 = moderate; 15 = moderate–severe; 20 = severe depression. Cut-off = 10) ISI	A significant reduction in anxiety (*t* (85)=3.51, *p* = 0.001, *d* = 0.38), depression (*t* (78):3.30, *p* = 0.001, *d* = 0.37) and insomnia (*t* (83)=3.95, *p* < 0.001, *d* = 0.43) was found.	According to the version of the GAD reported in the study, a total recovery on anxiety was found. According to the version of the PHQ reported in the study, a total recovery on depression was found.
Brennstuhl, 2022 (42) France	Pre-post without control group. Recruitment: July, 1, 2020 to August 30, 2020.	*N* = 21 (45.1 years old; *SD* = 11.1) 52.4% women, 81% in a relationship.	Severe cases of COVID-19. Patients admitted to ICU.	Anxiety or depression.	EMDR, 4 sessions. Antidepressants (19%), anxiolytics (23.8%), both (38.1%). No medication (19%)	Anxiety and depression: HADS-A HADS-D Multidimensional Assessment of COVID-19-Related Fears (MAC-RF). Score range = 0–32. Higher scores indicate higher fear levels.	Significant improvements were found in HADS-A (pre-test = 17.1; post-test = 10.8; X^2^ = 33.4, ddl 2, *p* < 0.001), HADS-D (pre-test = 14.6; post-test = 12.6; X^2^ = 9.5, ddl 2, *p* < 0.01) and MAC-FR (pre-test = 23.8; post-test = 13.09; X^2^ = 33.2, ddl 2, *p* < 0.001).	According to the original version of the HADS, a partial recovery on anxiety and depressive symptoms was found. According to the cut-off for MAC-RF, a total reduction of COVID-19 fears was found.
Cengiz, 2021 (43) Turkey	Single-center RCT NCT04696562 Recruitment: January to April 2021	*N* = 44 (Control = 22; intervention = 22), 51.64 years old (*SD* = 14.16), 52.3% female, 81.8% married, 50.0% primary studies.	COVID-19 patients treated in a hospital.	Anxiety.	Intervention: Deep breathing with Triflo: patients were sent a video to their mobile phone with training information about how to practice deep breathing with Triflo. Participants were encouraged to practice 5–10 times an hour until they went to sleep. Participants received two support calls a day. Control group: routine care from the hospital. No pharmacotherapy reported.	BAI: Scores range = 0–63. Higher scores indicate higher anxiety. World Health Organization Quality of Life Instrument Short Form (WHOQOL-Bref). Face-to-face and telephone.	In the experimental group, significant improvements were found in quality of life (pre = 74.05, *SD =* 7.42 – post = 77.82, *SD* = 6.77; Wilcoxon test = −3.74, *p* < 0.001) and anxiety (pre = 25.32, *SD* = 12.36 – post = 14.50, *SD* = 7.41; Wilcoxon test = −4.02, *p* < 0.001). In the control group, significant improvements were found in quality of life (pre = 62.50, *SD* = 15.97 – post = 65.95, *SD* = 14.54, Wilcoxon test = −2.94, *p* = 0.003) and anxiety (pre = 26.05, *SD* = 10.30 – post = 19.95, *SD* = 13.02; Wilcoxon test = −3.00, *p* = 0.003). Difference between the two groups at post assessments was not statistically significant (*p* > 0.050).	According to the original version of the BAI, a partial recovery was found in the experimental and control groups in anxiety symptoms.
Compagno, 2022 (44) Italy	Pre-Post without control group. Recruitment: April 2021–November 2021.	*N* = 30, 58.37 years old (SD = 11.6), 60% male.	Patients with long COVID syndrome. COVID-19 treatment: 53.3% hospital department; 16.6% ICU; 30% home.	Anxiety, depression.	4 sessions according to the specific symptoms of the patients: CBT, EMDR, muscular and imaginative relaxation, body-scan, breath control. Physical training programs: 3 training sessions per week of 90 min duration. No pharmacotherapy reported.	SDS: Scores range from 20 to 80 SAS: Scores range from 20 to 80	An improvement was observed in SAS (pre-test = 39.59, SD = 8.98 – post-test = 34.22; SD = 8.5; *p* < 0.05) and SDS scores (pre-test = 40.45, *SD* = 8.6 – post-test = 36.27, *SD* = 8.5; *p* < 0.05).	According to the cut-off reported in the study, participants had non-clinical symptoms of anxiety and depression before the intervention (scores on SAS and SDS below 45 and 50 points).
Fan, 2021 (45) China	Multicentric RCT (3 hospitals), ChiCTR2000039369. Recruitment: February 2020 to June 2020.	*N* = 111, 46.38 years old (*SD* = 12.34), 62.16% female, 92% married, 65% above middle school. Control group: Personalized psychological intervention =55 Intervention group: Narrative Exposure Therapy (NET) = 56	Patients recruited in 3 hospitals: 79.28% mild COVID-19, 20.72% severe COVID-19 condition.	Post-traumatic stress symptoms (PTSS)	Experimental. Internet. NET: 8 weeks (1–2 sessions a week, 90–120 min each time): psychoeducation, constructing the life event timeline and starting the narration. WeChat (COVID-19 prevention and psychological nursing information every week). Control: personalized psychological treatment based on participants’ symptoms once a week (40–60 min each time) and online follow-up. No pharmacotherapy reported.	PCL-C. Scores range = 17–85, cut-off ≥50 SDS: Scores range = 20–80, higher scores indicate greater depression SAS: Scores range = 20–80, higher scores indicate greater anxiety. PQSI: Scores range = 0–21, higher scores indicate poorer sleep quality.	A significant decrease in PCL-C was found in the intervention (pre = 75.20, *SD* = 4.01 – post-test = 49.52, *SD* = 7.32) and in the control group (pre = 74.45, *SD* = 4.86 – post-test = 58.65, *SD* = 7.48; F(1,109) = 639.976, *p* < 0.001). Significant decrease in SDS from pre-test to post-test in the intervention (pre = 53.52, *SD* = 11.84 – post-test = 46.89, *SD* = 8.95) and in the control group (pre = 54.29, *SD* = 11.51 – post-test = 50.40, *SD* = 8.98; *F*(1,109) = 14.159, *p* < 0.001). Significant decrease in SAS from pre-test to post-test in the intervention (pre = 61.11, *SD* = 11.42 – post-test = 51.64, *SD* = 9.5) and in the control group (pre = 61.47, *SD* = 11.84 – post-test = 50.70, *SD* = 10.23; F(1,109) = 52.142, *p* < 0.001). Significant decrease in PQSI from pre-test to post-test in the intervention (pre = 15.87, *SD* = 2.85 – post-test = 13.16, *SD* = 2.87) and in the control group (pre = 15.84, *SD* = 2.86 – post-test = 14.31, *SD* = 2.86; F(1,109) = 30.519, *p* < 0.001). The main effects of group on SDS, SAS and PQSI were not statistically significant (*p* > 0.050).	According to the PCL-C cut-off used in this study, a total recovery on post-traumatic symptoms was found in the experimental group. A partial recovery was found in the control group. According to the original version of the SDS, a total recovery on depressive symptoms was found in the experimental group. A partial recovery was found in the control group. According to the original version of the SAS, a partial recovery in the experimental and control groups was found in anxiety symptoms. According to the PSQI version employed in this study a partial recovery on sleep quality was found on the experimental and the control groups.
Ganesan, 2022 (46) India	Non-randomized two-group study. Recruitment: May 2020 – October 2020	*N* = 569 (tele counseling = 516; control group = 53). 43% female, around 43 years old.	COVID-19 patients in isolation wards.	Anxiety.	One tele counseling session (10–15 min): breathing exercises, pleasurable activities, eat and rest during isolation, communication, needs prioritization.	GAD (<7 mild; 7–14 moderate, >14 severe)	Significant reduction in anxiety levels in the tele counseling group compared with the control group (*p* < 0.001).	According to the cut-off reported in the study, a partial recovery on symptoms was found.
Torbati, 2022 (47) Iran	RCT. No recruitment data are reported.	*N* = 30 men (15 = experimental; 15 = control group). 100% male, age = 20–49 years old.	COVID-19 patients after hospital discharge.	Anxiety Depression	DBT: 10 sessions in 5 weeks (9 min each session). Face-to-face	BAI BDI	A significant reduction was found in depression (pre = 33.20; post = 28.66) and anxiety (pre = 35.26; post = 30.53) in the experimental group. Significant differences between the experimental and the control group were found at post-test in depression (*F* = 60.77; *p* < 0.001) and anxiety (*F* = 33.93; *p* < 0.001).	According to the original version of the BDI, a partial recovery on depressive symptoms was found. According to the original version of the BAI, a partial recovery on anxiety symptoms was found.
Ghodrati-Torbati, 2022 (48) Iran	Quasi-experimental pre-post study with a control group. No recruitment data reported.	*N* = 30 (15 control group = 15; experimental = 15). All men, 20–49 years old, 40–66% single, 26.66–40% academic studies.	COVID-19 patients after hospital discharge.	Anxiety Depression	Face-to-face. Compassion: 10 sessions of 90 min (two sessions per week): psychoeducation, breathing exercises, empathy and self-criticism, emotional regulation systems, forgiveness training, awareness concept, imagining training, self-compassion. Control group: waiting list. No pharmacotherapy reported.	BDI: scores range = 0–63, higher scores indicate greater depression. BAI: scores range = 0–63, higher scores indicate greater anxiety.	Reduction in the experimental group on BDI (pre-test = 33.60, *SD* = 3.08; post-test = 28.80, *SD* = 2.27) and BAI scores (pre-test = 36.33, *SD* = 2.41; post-test = 29.66, *SD* = 1.95). There were statistically significant differences between the experimental and control groups in post-test scores (*p* < 0.05, eta squared = 0.87).	According to the original version of the BDI-II, a partial recovery on depression was found in the experimental group. According to the original version of the BAI, a partial recovery from anxiety was found in the experimental group. No recovery in the control group in anxiety nor depression.
Kim, 2020 (49) Korea	Pre-post without control group. Recruitment: February 2020–April 2020.	*N* = 33, 45 years old (*SD* = 18.34)	Hospitalized and isolated COVID-19 patients.	Anxiety (18%), depression (39%), insomnia (30%), suicidal ideation (9%)	2-week CBT-based intervention. Telephone sessions of 30 min: psychoeducation, cognitive reconstructions for irrational beliefs, guidance for fear of re-infection. Pharmacotherapy = 27% of participants.	HADS-A: Cut-off >8 HADS-D: Cut-off >8 ISI: Cut-off >8 Suicidal ideation: BDI item 9	Significant improvements were found at one week in HADS-A (baseline = 5 – one week = 4; *p* = 0.019), HADS-D (baseline = 5 – one week = 4; *p* = 0.027), and suicidal ideation (baseline = 9.1% – one week = 0%; *p* = 0.045).	According to the cut-off reported in the study, participants had non-clinical symptoms of anxiety, depression and insomnia before the intervention (scores on HAD and ISI below 8 points).
Kong, 2020 (50) China	Single center RCT. Recruitment: February 2020 to March 2020.	*N* = 26 (control = 13; intervention = 13)	COVID-19 hospitalized patients.	Anxiety Depression	Control group: basic care during hospitalization. Intervention: Psychological-Behavioral Intervention (PBI), 10 sessions: breathing exercise, psychosocial support (express feelings, comfort patients, information about COVID-19, relaxation, self-emotional management skills). No pharmacotherapy reported.	HADS-A and HADS-D. 0–7 = no symptoms; 8–10 = mild symptoms; 11–14 = moderate symptoms; 15–21 = severe symptoms. PSSS: 12–36 = low; 37–60 = moderate; 61–84 = high social support.	A significant reduction in HADS-A (pre = 12.62, *SD* = 2.66 – post = 6.15, *SD* = 3.58, t = 6.10, *p* < 0.001) and HADS-D (pre = 11.69, *SD* = 2.93 – post = 5.92, *SD* = 3.73, t = 5.88, *p* = 0.001) was found in the intervention group. No significant reduction in HADS-A (t = 1.94, *p* = 0.076) and HADS-D (t = 1.79, *p* = 0.098) was found in the control group. Significant differences in HADS-A and HADS-D scores (t = −2.68, *p* = 0.013) were found between the intervention and the control group. PSSS was improved in the intervention group (pre = 54.69, SD = 15.59 – post = 64.46, *SD* = 11.05, t = −4.96, *p* < 0.001), but not in the control group (t = −1.24, *p* = 0.241).	According to the cut-off of the HADS reported in this study, a total recovery on anxiety and depression was found in the intervention group. A partial recovery was found in the control group. According to the cut-off of the PSSS reported in this study, a total recovery on social support was found in the experimental and control groups.
Lerthattasilp, 2021 (51) Thailand	Prospective controlled study. Recruitment: March 2020 – May 2020.	*N* = 40 (intervention =21), mean age = 31.7 (*SD* = 10.4) 76.2% female; control = 19, 26.8 years old (*SD* = 6.1).	COVID-19 hospitalized patients.	Depression (15%), anxiety (30%)	“LINE” social messaging application: quarantine psychoeducation, stress management, breathing exercises, progressive muscle relaxation, meditation and exercise sessions. 3 online group video calls per week. Control group: participants who declined to join LINE. Participants did notreceive pharmacotherapy.	DASS-21: Depression >4 points. Anxiety >3 points. Stress >7 points.	In the intervention group, a reduction was found in depression BL = 3.6, *SD* = 5.0 – FU (BL = 4.4, *SD* = 4.8 – FU = 2.2, *SD* = 4.0) and stress (BL = 4.8, *SD* = 4.2 – FU = 3.5, *SD* = 4.2). In the control group, a reduction was found in depression (BL = 1.6, *SD* = 1.9 – FU1.2, *SD* = 2.0), anxiety (BL = 1.5, *SD* = 2.0 – FU = 0.8, *SD* = 1.4) and stress (BL = 2.4, *SD* = 2.5 – FU = 1.7, *SD* = 2.2).	According to the cut-off reported in the study, participants had non-clinical symptoms of depression and stress before the intervention (scores on DASS below 4 and 7 points respectively).
Li, 2020 (52) China	RCT, single center. Recruitment: February 2020 to March 2020.	*N* = 93 (control = 46; experimental = 47), 64.5% women, 54.4–57.5% employed, 63–68.1% secondary studies, 83–84.8% married.	Hospitalized in single isolation wards. Mild symptoms of COVID-19.	Depression (53.8%), anxiety (90.3%)	Experimental: routine treatment + CBT delivered by nurses: cognitive intervention, relaxation techniques, problem solving, social support. Face-to-face, daily, 30 min each session. Control: routine treatment including antiviral treatment, symptomatic treatment of fever and nursing care.	DASS-21: Depression: 0–9 = normal; 10–13 = mild; 14–20 = moderate; 21–27 = severe; >27 = extremely severe depressive symptoms. Anxiety: 0–7 = normal; 8–9 = mild; 10–14 = moderate; 15–19 = severe; >19 = extremely severe anxiety symptoms. Stress: 0–14 = normal; 15–18 = mild; 19–25 = moderate; 26–33 = severe; >33 = extremely severe stress symptoms.	Significant reduction in the experimental group was found in depression (pre = 11.0, *SD* = 3.30 – post = 7.98, *SD* = 2.42; mean difference = −3.06, *p* < 0.001), anxiety (pre = 17.10, *SD* = 4.4 – post = 10.30, *SD* = 3.70; mean difference = −6.81, *p* < 0.001) and stress (pre = 16.8, *SD* = 3.59 – post = 13.1, *SD* = 3.44; mean difference = −3.72, *p* < 0.001). Significant reduction in the control group was found in depression (pre = 10.10, *SD* = 3.17 – post = 8.07, *SD* = 2.16; mean difference = −2.00, *p* = 0.001), anxiety (pre = 16.50, *SD* = 4.81 – post = 11.20, *SD* = 3.67; mean difference = −5.33, *p* < 0.001) and stress (pre = 17.10, *SD* = 3.71 – post = 12.80, *SD* = 2.47; mean difference = −4.28, *p* < 0.001).	According to the cut-off of the DASS-21 reported in this study, a total recovery from depression was found in the experimental and control groups. A partial recovery from anxiety was found in the experimental and control groups. A total reduction on stress was found in the experimental and control groups.
Liu, K. 2020 (53) China	RCT, single center. Recruitment: January 2020 to February 2020.	*N* = 51 (control = 26; experimental =25), 50.41 years old (*SD* = 13.04), 53.85–56% men.	COVID-19 patients admitted to a hospital	Anxiety	Experimental: Jacobson’s relaxation techniques (progressive muscle relaxation and deep breathing), 20–30 min per day during 5 consecutive days. Control: routine care. No pharmacotherapy reported.	STAI: ≤20 = no symptoms; 21–39 = mild; 40–59 = moderate; 60–80 = severe. Sleep State Self-Rating Scale (SRSS): scores between 10 (no sleeping problems) and 50 (severe sleeping problems)	Significant differences in STAI scores were found between the experimental (pre = 24.04, *SD* = 3.87 – post = 16.76, *SD* = 4.10) and the control group (pre = 23.85, *SD* = 2.82 – post = 23.23, *SD* = 2.70; *p* < 0.001). Significant differences in SRSS were found between the experimental (pre = 57.88, *SD* = 11.51 – post = 44.96, *SD* = 12.68) and the control group (pre = 56.92, *SD* = 7.92 – post = 57.15, *SD* = 9.24; *p* < 0.001)	According to the cut-off of the STAI reported in this study, a total recovery on anxiety symptoms was found in the experimental group. Mild symptoms of anxiety remained stable in the control group.
Liu Y, 2021 (54) China	RCT, single center. Recruitment: March 2020	*N* = 140 (Control =70; intervention = 70), 40–44% >45 years old, 52.86–65.71% female, 74.29–84.29% married, 44.29–55.71% senior school and below, 64.29–70.00% employed.	Mild COVID-19 patients.	Anxiety Insomnia	Control: medical routine care for COVID-19. Intervention: psychological intervention and pulmonary rehabilitation (breathing exercises, music therapy and pulmonary rehabilitation training). WeChat group as a communication platform: realistic information about COVID-19, social support, spiritual demands, lectures and positive suggestions by psychological experts, baduanjin exercises. Some patients received individual psychological intervention. No pharmacotherapy was provided.	STAI-S, total scores 20–80 points, greater scores indicate more severe anxiety symptoms. PSQI >7 possible sleeping problems.	The intervention group showed an increased reduction in STAI-S (mean = 38.5, *SD* = 13.2) compared with the control group (mean = 45.8, SD = 10.4, t = 3.60, *p* < 0.001) The intervention group showed an increased reduction in PSQI (mean = 5.6, *SD* = 3.0) compared with the control group (mean = 7.1, *SD* = 3.0, t = 2.98, *p* = 0.003).	According to the cut-off of the PSQI reported in this study, a total recovery on insomnia was found on the intervention group.
Liu Z, 2021 (55) China	RCT, multicenter (5 hospitals) ChiCTR2000030084 Recruitment: March 2020–June 2020.	*N* = 252 (Control = 126, experimental = 126), 55.55–63.49% men, 41.5–43.7 years old, 10.6 years of education.	Mild or common type of COVID-19 patients.	Anxiety Depression	Experimental: computerized CBT program (mobile, iPad): minimize negative thoughts about COVID-19, relaxation mental imagery training, mindfulness meditation. 10 min of individual therapy per day for 1 week. Control: psychological assessment, general psychological support and consultations about overall well-being and disease activity. Participants did not take pharmacotherapy.	HAMD: Scores ≥7 indicate mild–moderate symptoms. HAMA: Scores ≥7 indicate mild to moderate symptoms. Athens Insomnia Scale (AIS): <4 = no insomnia; 4–6 = suspicious symptoms, >6 = insomnia.	The experimental group showed significant improvement in HAMD (pre = 15.13, *SD* = 3.33 – post = 8.19, *SD* = 3.54, *p* < 0.001, *d* = 2.02), HAMA (pre = 14.52, *SD* = 3.13 – post = 7.79, *SD* = 3.60, *p* < 0.001, *d* = 1.97), and AIS (pre = 8.98, *SD* = 3.45 – post = 7.52, *SD* = 2.99, *p* < 0.001, *d* = 0.63). No significant differences were found in the control group in HAMD (pre = 15.52, *SD* = 3.43 – post = 15.20, *SD* = 3.64, *p* = 0.080, *d* = 0.16), HAMA (pre = 13.97, *SD* = 2.72 – post = 13.63, *SD* = 3.24, *p* = 0.120, *d* = 0.14), nor AIS (pre = 8.67, *SD* = 3.08 – post = 8.27, *SD* = 3.22, *p* = 0.070, *d* = 0.16).	According to the cut-off of the HAMD reported in this study, a partial recovery on depressive symptoms was found in the experimental group. According to the cut-off of the HAMA reported in this study, a partial recovery on anxiety symptoms was found in the experimental group. According to the cut-off of the AIS reported in this study, a partial recovery on insomnia was found in the experimental group. The control group did not recover from the anxiety, depression and insomnia symptoms.
Mahendru, 2021 (56) India	RCT. Recruitment: June 2020–July 2020.	*N* = 84 (control = 42; experimental = 42), 34.52–36.48 years old, 66–69% males, 19–31 graduate studies	Asymptomatic or mildly symptomatic COVID-19 infected patients	Depression Anxiety Insomnia	Intervention: Meditation and breathing exercises through videos sent on WhatsApp. Participants are encouraged to meditate 5–10 min 3 times a day for 7 days. No pharmacotherapy reported.	DASS-21: Depressive symptoms >9 scores. Anxiety symptoms >7 scores Stress>14 scores. Quality of sleep: *ad hoc* items.	At post-test significant differences were found between the control and intervention groups in depression (mean control = 4.67, mean intervention = 1.81, *p* < 0.001) and stress (mean control = 4.25, mean intervention = 2.71, *p* = 0.004). No significant differences were found in anxiety (mean control = 1.81, mean intervention = 2.14, *p* = 0.528). The quality of sleep and feeling tired after waking up in the morning were also better in the intervention group (*p* < 0.050).	According to the cut-off of the DASS-21 reported in this study, a total recovery from depression, anxiety and stress was found in both groups.
Maresca, 2022 (57) Italy	Quasi-experimental one group pretest-posttest Recruitment: March 2020–June 2020	*N* = 45, 44.2 years old (*SD* = 14.4), 42.2% women.	COVID-19 patients.	Depression and insomnia (97.8%) Anxiety (68.9%) 66.7% presented all three conditions.	“Telecovid Sicilia,” web-based platform. Individual 1-h sessions twice a week, total of 16 sessions: relational systemic approach: management and resolution of psychological symptoms derived from the COVID-19 and isolation. No pharmacotherapy was provided.	SCL-90-R: anxiety, depression and paranoid ideation. BDI Epworth Sleepiness Scale (ESS) HAMA	A significant reduction was found in BDI (baseline = 18.0 – follow-up = 12.0, *p* < 0.001, effect size = 0.617), EES (baseline = 11.0 – follow-up = 7.0, *p* < 0.001, effect size = 0.618), and HAMA (baseline = 18.0 – follow-up = 11.0, *p* < 0.001, effect size = 0.618).	According to the original version of the BDI-II, a total recovery on depressive symptoms was found. According to the original version of the HAMA, a total reduction on anxiety symptoms was found. According to the original version of the EES, a total reduction on sleepiness was found.
Parizad, 2021 (58) Iran	Single-blinded, parallel RCT. Recruitment: June 2020–July 2020.	*N* = 62 (control = 32; intervention = 30), 37.32–43.14 years old, 54.5–58.2% male, 34.5–52.7% high school, 67.3–76.4% married	Non-hospitalized COVID-19 patients.	Anxiety	Intervention: Guided imagery training, patients should imagine controlling horrific events. In each session, five audio tracks were administered by the nurse, and the patient listened to the instructional guided imagery audio tracks for about 25 min. Ten sessions for five consecutive days (twice a day). Control group: routine care. No pharmacotherapy was reported.	STAI: Scores range = 20–80. Low scores = mild anxiety; middle scores = moderate anxiety; high scores = severe anxiety.	No significant differences were found in the control group in STAI-S (pre = 46.72 – post = 47.21; t = −1.259, *p* = 0.214, *d* = 0.16) and STAI-T (pre = 46.47 – post = 46.00; t = 0.487, *p* = 0.629, *d* = 0.06). Significant differences were found in the experimental group in STAI-S (pre = 45.03 – post = 38.27, t = 8.161, *p < 0*.001, *d* = 1.10) and STAI-T (pre = 47.34 – post = 39.58, t = 7.962, *p < 0*.001, *d* = 1.07).	According to the cut-off of the STAI reported in this study, a partial recovery from anxiety was found.
Priyamvada, 2021 (59) India	Pre-post design No recruitment data.	*N* = 30, 80% were 20–40 years old, 83% male, 80% graduate, 93% married	Recovered COVID-19 patients.	Anxiety Depression	Psychoeducation, breathing exercises, autogenic training (change negative views by positive affirmative statement), activity scheduling, social support and emotion regulation strategies. 30 min twice a week for a month, total of 8 sessions. No pharmacotherapy was reported.	Mental Health Inventory (MHI): anxiety, depression, behavioral control and positive affect. Scores for all subscales range between 1 and 6. Higher scores indicate greater mental health.	Significant differences were found in anxiety (pre = 2.96 – post = 5.23, Wilcoxon = −4.71, *p* < 005), depression (pre = 3.03 – post = 5.26, Wilcoxon = −4.69, *p* < 005), behavioral control (pre = 3.40 – post-5.63, Wilcoxon = −4.60, *p* < 005) and positive affect (pre = 2.96 – post = 5.61, Wilcoxon = −4.69, *p* < 005)	The original version of the MHI does not provide cut-off scores.
Rutkowski, 2022 (60) Poland	RCT. No recruitment data.	*N* = 32 (VR = 16; control = 16). 68.75% female, age = 57.8 years old.	COVID-19 patients cared for in an inpatient rehabilitation program.	Anxiety Depression	3-week rehabilitation program, five times a week. Rehabilitation program combined with mental health support (Ericksonian psychotherapy).	HADS: Cut-off = 8 points. WHO Quality of life-BREF	Both groups showed a reduction in anxiety and depressive symptoms. Anxiety experimental (pre = 8.6; post = 5.6; *p* < 0.001), depression experimental (pre = 6.9; post = 4.7, *p* = 0.008). Anxiety control (pre = 9.57; post = 8, *p* = 0.003), depression control (pre = 7.64; post = 6.6, *p* = 0.017).	According to the cut-off reported in the study, both groups showed a total recovery from anxiety and depressive symptom.
Shaygan, 2023 (61) Iran	RCT. Recruitment: 2020.	*N* = 72 (experimental = 36; control =36). 47–68% female, 30–50 years old.	COVID-19 patients in home quarantine.	Anxiety	WhatsApp group to provide videos, audio and educational text with coping strategies, positive thinking, spiritual well-being and relaxation music. Daily sessions over 14 days.	Online assessments STAI BAI	Significant differences were found between groups in state (experimental = 34.69; *SD* = 10.75; control = 45.75, *SD* = 13.01; *F* = 16.52; *p* < 0.001) and trait anxiety (experimental = 38.31; control = 46.50; *t* = −2.49; *p* = 0.010).	According to the original version of the STAI, a partial recovery from anxiety symptoms was found (scores at post-test)
Sotoudeh, 2020 (62) Iran	RCT, single-center. Recruitment: May–June 2020.	*N* = 30 (control = 14, experimental = 16), 53.3% female, 20–70 years old, 63.4% married, 46.6% high school diploma or less.	COVID-19 patients.	Stress Depression Anxiety	Experimental: Brief Crisis Intervention. 4 sessions: relaxation, adjustment techniques, resilience to COVID-19, tension reduction, cognition and meta-cognition. Control group: standard individual psychotherapy. No pharmacotherapy was reported.	DASS-21 WHO-QOL-BREF	Scores for depression in the experimental group (pre = 14.5, *SD* = 4.5 – post = 12.1, *SD* = 3.4) were lower than in the control group (pre = 12.2, *SD* = 3.6 – post = 9.05, *SD* = 2.1; *p* = 0.010). Scores for anxiety in the experimental group (pre = 17.5, *SD* = 4.8 – post = 11.7, *SD* = 3.5) were lower than in the control group (pre = 14.2, *SD* = 4.2 – post = 9.10, *SD* = 3.2; *p* = 0.030). Lower stress levels were found in the experimental group (pre = 20.2, *SD* = 4.1 – post = 15.1, *SD* = 4.2) compared with control group (pre = 13.8, *SD* = 4.5 – post = 10.3, *SD* = 3.3, *p* = 0.020). Improvements in quality of life were found in the experimental group (pre = 69.2, *SD* = 13.1 – post = 85.1, *SD* = 15.7) compared with the control group (pre = 76.5, *SD* = 9.7 – post = 82.9, *SD* = 11.3, *p* = 0.030).	According to the original version of the DASS-21, a partial recovery from depressive and anxiety symptoms was found in both groups.
Sun, 2021 (63) China	Pre-post without control group. Recruitment: January 2020–March 2020.	*N* = 97, 51.55% female.	COVID-19 patients (*N* = 71) in the isolation ward.	Anxiety	Patients with normal and mild anxiety received 1–2 sessions a week. Patients with moderate and severe anxiety received 2–3 sessions a week. No pharmacotherapy was reported.	SAS: normal, mild, moderate or severe symptoms.	The SAS score from patients was significantly lower on the 14th day of isolation (mean = 63.42, *SD* = 8.86) than on the 7th day (mean = 73.81, *SD* = 9.71, *p* < 0.01).	According to the original version of the SAS, a partial recovery was found from anxiety symptoms.
Wei, 2020 (64) China	RCT, single center. Recruitment: February 2020	*N* = 26 (control = 13, experimental = 13)	COVID-19 patients in the isolation ward.	Anxiety Depression	Experimental: Internet-based program with audios: breath relaxation, mindfulness, refuge skills and butterfly hug method. Daily 50-min sessions for 2 weeks. No pharmacotherapy was reported.	PHQ-9 ≥ 5 HAMD-17 GAD-7 ≥ 5 HAMA	HAMD and HAMA were significantly decreased in patients in the intervention group at week 1 (17-HAMD, t = −2.381, *p* = 0.026; HAMA, t = −2.263, *p* = 0.033) and week 2 (17-HAMD, t = −3.089, P = 0.005; HAMA, t = −3.746, P = 0.001) when compared with the patients in the control group	Authors do not report means at post-test. It is not possible to establish whether a partial or total recovery was found.
Won, 2023 (65) Korea	Pre-post without control group. Recruitment: March 2020–April 2020	*N* = 32 (positive screening = 21; negative screening = 11). 50.56 years old, 62% women.	COVID-19 admitted to the inpatient ward.	Depression Anxiety PTSD	Telephone counseling, no information about the psychological intervention.	Online surveys: PHQ-9: Cut-off = 6 GAD-7: 5–9 mild; 10–14 moderate; >15 severe anxiety. PC-PTSD-5: Cut-off = 3	There were no significant improvements in the PHQ-9 and GAD-7 (*p* > 0.050). A significant reduction in PTSD symptoms was found (*p* = 0.041).	According to the cut-off reported in the study for the PC-PTSD, participants presented non-clinical scores before the intervention.
Xiao, 2020 (66) China	Patients were assigned to one of two groups, according to their wishes. Recruitment: February 2020 – March 2020	*N* = 79 (observation =39; control =40)	COVID-19 patients hospitalized for more than 7 days.	Anxiety Depression Insomnia	Observation: Progressive muscle relaxation in bed, 30 min before getting up early and 30 min before going to bed for 1 week. Each session lasted 15 min. Patients were trained through videos and explanations from professionals. Control: instruction to perform body movement in bed. No pharmacotherapy was reported.	GAD-7: Scores ≥5 indicates anxiety. PHQ-9: Scores ≥5 indicates depression. PSQI: Scores ≥8 indicates poor sleep quality.	The observation group showed significant lower GAD-7 (pre = 5.38, *SD* = 5.25 – post = 3.69, *SD* = 2.99) compared with the control group (pre = 5.72, *SD* = 3.71 – post = 5.77, *SD* = 3.72, *t* = −2.74; *p* = 0.008). The observational group showed lower PHQ-9 (pre = 5.05, *SD* = 4.86 – post = 3.69, *SD* = 3.93) compared with the control group (pre = 5.20, *SD* = 2.88 – post = 6.02, *SD* = 3.74, *t* = −3.04; *p* = 0.003). The observational group showed lower PSQI (pre = 10.25, *SD* = 2.75 – post = 7.41, *SD* = 2.42) compared with the control group (pre = 10.08, *SD* = 5.43 – post = 9.72, *SD* = 5.08, *t* = −2.57; *p* = 0.012).	According to the cut-off of the GAD-7 reported in this study, a total recovery from anxiety was found in the experimental group. According to the cut-off of the PHQ-9 reported in this study, a total recovery from depression was found in the experimental group. According to the cut-off of the PSQI reported in this study, a total recovery from insomnia was found in the experimental group. The control group did not recover from anxiety, depression and insomnia symptoms.
Yang, 2020 (67) China	Pre-post without control group. Recruitment: February 2020–March 2020	*N* = 35, 57 years old (*SD* = 13.5), 60% men, 85.71 married, 69.60% high school.	COVID-19 patients isolated in the ICU.	Anxiety Depression Insomnia	Face-to-face and online sessions: supportive, psychotherapy, empathy, muscle and breath relaxation, CBT (case formulation and recognition of emotions). Sessions of 15–30 min, three times a week. No pharmacotherapy was reported.	PSQI: 0–5 = very good night’s sleep; 6–10 = sleep quality not bad; 11–15 = sleep quality fairly bad; 16–21 = sleep quality very bad. PHQ-9: <4 = minimal; 5–9 = mild; 10–14 = moderate; 15–19 = moderately severe; 20–27 = severe. GAD-7: 0–4 = minimal; 5–9 = mild; 10–14 = moderate; >15 = severe. Social Support Rate Scale (SSRS): scores range = 12–66. Greater scores mean higher satisfactory social support.	Significant improvements were found after the intervention in SRSS (BL = 25.57 – FU = 29.94, *p* < 0.001), PSQI (BL = 11.20 – FU ≈ 5.0, *p* < 0.001), PHQ-9 (BL = 8.80 – FU ≈ 4, *p* < 0.001), and GAD-7 (BL = 10.69 – FU ≈ 4, *p* < 0.001).	According to the SRSS version used in this study, a partial recovery on social support was found. According to the cut-off of the PSQI reported in this study, a total recovery from insomnia was found. According to the cut-off of the PHQ-9 reported in this study, a total recovery from depressive symptoms was found. According to the cut-off of the GAD-7 reported in this study a total recovery on anxiety was found.
Yuan, 2021 (68) China	Experimental design with a control group. Recruitment: February 2020–March 2020	*N* = 65 (Experimental = 31; control = 34), 42–52% female, 55.91–56.77 years old.	COVID-19 patients admitted to a hospital.	Anxiety Depression	Experimental: WeChat group between medical staff and patients: health education and rehabilitation training guidance. Patients received videos and written materials about medication, diet and psychological counseling. Control group: routine treatment. No pharmacotherapy was reported.	HADS: 0–7 = asymptomatic; 8–10 = suspicious; 11–21 = definitely present. “Suspicious” and “symptomatic” are positive patients. PANAS: higher scores indicate greater positive and negative affect. Coping Modes Questionnaire: confrontation, avoidance and acceptance-resignation.	There were no significant differences in coping styles between the experimental and control groups (*t* = 1.18, *p* = 0.241). The experimental group obtained a significant reduction in PANAS (mean = 19.58, *SD =* 6.61) compared with the control group (mean = 24.53, SD = 7.44, t = −2.82, *p* = 0.006). The experimental group obtained a significant lower HADS (mean = 11.71, *SD* = 3.64) than those of the control group (mean = 15.44, *SD* = 3.86, t = −4.00, *p* < 0.001).	According to the cut-off of the HADS reported in this study, a partial recovery from anxiety symptoms was found on the experimental group. The control group did not recover from symptoms.
Zheng, 2022 (69) China	RCT No recruitment data.	*N* = 70 (control = 35; observation = 35), 31–37 years old.	COVID-19 patients.	PTSD Anxiety	Observation: positive therapy and deep hypnosis to increase patient tolerance to fear-sensitive stimulation. Professionals determined the frequency and time of the intervention according to the physical and mental health status of the patients. Control: routine rehabilitation therapy, symptomatic psychological counseling, pharmacotherapy.	SAS: <4 = mild; 4–7 = moderate; >7 = severe. SDS	Significant differences between the control (mean = 5.28, *SD* = 0.63) and observational groups (mean = 4.33, *SD* = 0.54) were found in SAS (t = 5.24, *p* = 0.007). Significant differences between the control (mean = 5.34, *SD* = 0.71) and observational groups (mean = 4.58, *SD* = 0.65) were found in SDS (t = 6.38, *p* = 0.008).	According to the cut-off of the SAS reported in this study, a partial recovery from anxiety was found in both groups.

*We have included only the first authors’ name. GAD-7: Generalized Anxiety Disorder Assessment; PHQ-9: Patient Health Questionnaire; ISI; Insomnia Severity Index; ICU: Intensive Care Units; EMDR: Eye Movement Desensitization and Reprocessing Therapy; HADS: Hospital Anxiety and Depression Scale; BAI: Beck Anxiety Inventory; SDS: Self-Rating Depression Scale; SAS: Self-Rating Anxiety Scale; NET: Narrative Exposure Therapy; PCL-C: PTSD Checklist-Civilian Version; PQSI: Pittsburgh Sleep Quality Index; BDI: Beck Depression Inventory; PSSS: Perceived Social Support Scale; DASS-21: Depression Anxiety and Stress Scale-21; BL: baseline; FU: follow-up; STAI: Spielberg State–Trait Anxiety Scale; PSQI: Pittsburgh Sleep Quality Index; HAMD: Hamilton Depression Rating Scale; HAMA: Hamilton Anxiety Scale; SCL-90-R: Symptom Checklist-90-R; VR = Virtual Reality; PANAS: Positive and Negative Affect Schedule.

### 2.4 Risk of bias assessment

The analyses of the quality of the studies were performed by two independent researchers (*VM-B and LM-G*). We did not exclude any articles due to their study design (i.e., controlled intervention studies, observational cohort and cross-sectional studies, case–control studies, before-after with no control studies and case series studies). Consequently, the Study Quality Assessment Tool that was developed by the National Heart, Lung and Blood Institute (70) was employed.

## 3 Results

**Figure 1:**
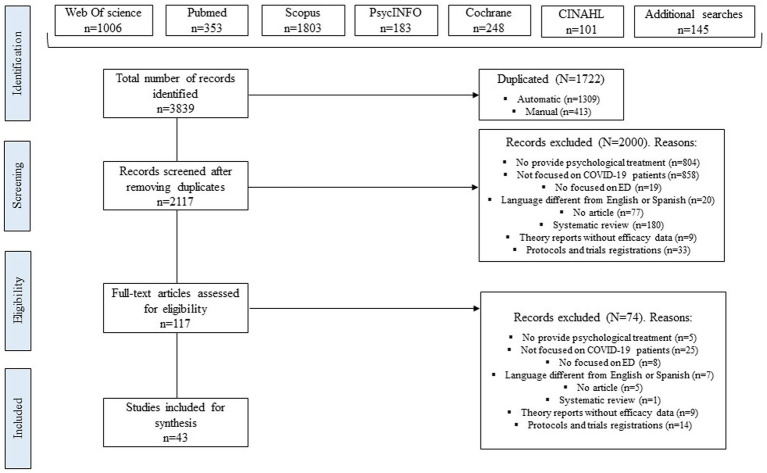
PRISMA flow diagram of included studies (23).

As can be observed in Figure 1, a total of 3,839 records were identified from electronic searches on databases and additional searches on references of systematic reviews. Of those, 2,117 were kept after eliminating duplicated records. In the first phase of screening, 2000 were excluded looking at title and abstract; the most frequent exclusion criteria were a psychological intervention was not provided (*n* = 804) or the target intervention did not include COVID-19 patients (*n* = 858). In the second phase, a total of 117 full-text articles were assessed for eligibility and only 43 were included in this review for synthesis. Agreement between the two independent researchers in the selection of the studies was 98% (Cohen’s *k* = 0.75, substantial agreement).

### 3.1 Descriptive characteristics of studies included

Characteristics of the 43 scientific studies included in this systematic review are reported in Table 1 (case studies) and Table 2 (intervention studies with and without control group). Sample size in the different studies ranged from 1 to 569. Across all the studies, a total of 2,359 participants were included. With regard to the age of participants, it ranged from 20 to 72 years old. Some studies (*n* = 6) did not provide participant’ age information (33, 50, 52, 63, 64, 66).

Most of the studies had been conducted in China (*n* = 15) (30, 33, 34, 45, 50, 52–55, 63, 64, 66–69), Iran (*n* = 6) (38, 47, 48, 58, 61, 62), Italy (*n* = 5) (29, 31, 41, 44, 57), India (*n* = 4) (36, 46, 56, 59), United States (*n* = 2) (28, 35), and Korea (*n* = 2) (49, 65). The remaining studies had been developed in Saudi Arabia (*n* = 1) (27), Nigeria (*n* = 1) (32), Indonesia (*n* = 1) (39), France (*n* = 1) (42), Turkey (*n* = 1) (43), Thailand (*n* = 1) (51), Latvia (*n* = 1) (40), Ghana (*n* = 1) (37), and Poland (*n* = 1) (60).

With regard to study design, the most common study design was randomized controlled trials (*n* = 15) (43, 45, 47, 50, 52–56, 58, 60–62, 64, 69) followed by case studies (*n* = 14) (27–40). Other study designs included pre-post studies without control group (*n* = 9) (41, 42, 44, 49, 57, 59, 63, 65, 67) and non-randomized studies with control group (*n* = 5) (46, 48, 51, 66, 68).

### 3.2 COVID-19 characteristics

In relation to COVID-19 characteristics, as presented in Tables 1, 2, out of the total 43 studies, only three studies (two case studies and one pre-post study without control group) included long COVID-19 patients (28, 40, 44) while the remaining investigations were focused on COVID-19 patients.

The difference in the severity of COVID-19 symptoms is clearly appreciated across the studies. On the one hand, some studies focused on mildly-affected patients, namely participants who had recovered from COVID-19 symptoms (*n* = 5) (31, 32, 38, 47, 59), who were asymptomatic (*n* = 2) (39, 56), presented mild COVID-19 symptoms (*n* = 2) (54, 55) or were out of hospital (*n* = 3) (48, 58, 61). On the other hand, other interventions had been provided to COVID-19 patients who were hospitalized in isolation wards (*n* = 20) (29, 30, 33–37, 43, 46, 49–53, 60, 63–66, 68) or patients with severe symptoms in Intensive Care Units (*n* = 3) (42, 66, 67). Additional researches recruited participants from various settings and with different levels of severity of COVID-19 symptoms (*n* = 3) (41, 45) or did not specify the severity of the COVID-19 symptoms (*n* = 3) (27, 57, 69).

### 3.3 Characteristics of psychological interventions

Regarding emotional disorders or symptoms addressed, the vast majority of studies were focused on depressive symptoms alone (30) or combined with anxiety (*n* = 15) (33, 34, 40–42, 44, 47, 48, 50–52, 55–57, 60), panic attacks (*n* = 6) (59, 62, 64, 66–68), suicidal ideation or self-harming attempts (*n* = 2) (36, 37) or post-traumatic stress disorder (PTSD) (*n* = 1) (38). On the other hand, six studies were focused on patients who presented with anxiety symptoms alone (43, 46, 53, 54, 58, 61) or combined it with panic attacks (29, 35).

The remaining studies addressed panic attacks alone (63) or combined with PTSD (69), PTSD alone (28, 31, 45), or obsessive-compulsive disorder (OCD) (27). Finally, two studies included patients with more than two diagnoses, namely depression, anxiety, panic attacks and suicidal attempts (39), depression, anxiety and suicidal attempts (32, 49) or depression, anxiety and PTSD (65).

Different psychological approaches were employed to manage the aforementioned psychological issues. Some studies (*n* = 10) were based on cognitive and behavioral principles [CBT alone (27, 49, 52); CBT + eye movement desensitization and reprocessing (EMDR) (44); CBT + mindfulness (55); CBT + relaxation (67); Cognitive Processing (28); Behavioral Therapy (50); dialectical-behavioral therapy (DBT) (34, 47)].

Other interventions had an interpersonal / relation approach (*n* = 4) [Interpersonal Therapy (33); emotion focused therapy based on interpersonal relationships (38, 41); relational intervention (57)]. Additional interventions included relaxation alone (*n* = 3) (53, 62, 66) or combined with mindfulness (64). Three studies used breathing techniques as the main component (29, 43, 56) while six interventions were based on providing psychoeducation, social support and additional relaxation and meditation techniques (46, 51, 54, 59, 61, 68).

The interventions with less representation were those based on music therapy (39, 40); compassion (48); positive psychology with hypnosis and Ericksonian principles (60, 69), narrative exposure therapy (45), EMDR (31, 42) and imagination (58). Four case studies (30, 32, 35, 36) and two pre-post studies without control group (63, 65) did not provide information about the type of psychotherapy that was applied. One study (37) described two different psychological programs, one of them based on CBT and the other focused on social support, positive recovery and relaxation training.

In terms of programs’ length, as shown in Tables 1, 2, short and long interventions were used. Some programs were implemented in only 1–5 sessions (33, 34, 39, 42, 53, 62) while others lasted 8–12 sessions (28, 31, 41, 45, 47, 48, 50) or had over 16 appointments (29, 57). The frequency of sessions also showed great variability. Some studies offered daily (36, 43, 52, 61) or weekly sessions (28, 31) while others proposed 2–3 sessions per week (44, 48, 57, 67). Again, some inconclusive results were found in the sessions duration, which ranged from 5–10 min (46, 47, 55, 56) to 60–90 min (44, 45, 48) (Tables 1, 2).

With regards to the format, some interventions had face-to-face appointments (47, 48, 52, 66) sometimes combined with online sessions (67). Other interventions used technology to provide the entire intervention. For example, seven studies used computerized programs which required the use of Internet-based solutions (45, 55, 57, 64) or videoconferencing (29, 36, 41). Four programs used group social messaging platforms (51, 54, 61, 68) and three studies used phone calls (35, 49, 65). Finally, some interventions were supported by the use of videos and audios (43, 56, 58, 66).

### 3.4 Intervention efficacy

Different measures were used across studies to assess changes in psychological outcomes after the intervention. Instruments used to assess depressive symptoms included the PHQ (28, 29, 33, 40, 41, 64–67), HAMD-D (33, 34, 55, 64), the BDI (38, 47–49, 57), the HADS-D (42, 49, 50), the SDS (44, 45, 69) and the MADRS (34). Similarly, seven different instruments were used to assess anxiety symptoms, namely, HAMA (33, 34, 55, 57, 64), GAD (28, 29, 41, 46, 64–67), HADS-A (42, 49, 50, 60, 68), STAI (53, 54, 58, 61), BAI (43, 47, 48, 61), and SAS (44, 45, 63, 69). As can be seen in Table 2, some authors assessed both anxiety and depressive symptoms with two different instruments while others selected one isolated measure, such as the DASS, the SCL-90-R or the Mental Health Inventory, which includes the assessment of multiple outcomes (51, 52, 56, 57, 59, 62, 65).

Another outcome that was assessed in various studies was insomnia with instruments as the PQSI (45, 54, 66, 67), SRSS (53, 67), ISI (29, 49), AIS (55), ESS (57) or *ad hoc* questions (56). Additional outcomes assessed were posttraumatic symptoms (PCL-C) (28, 38, 45, 65), quality of life (WHOQOL) (43, 60, 62), social support (PSSS) (50), affect (PANAS) (68), and coping (Coping Modes questionnaire) (68). It is also remarkable that only one study used a COVID-19 specific measure, namely the MAC-RF, to assess COVID fears (42). The rest of the studies used clinical interviews (30, 32, 35), or did not inform about how they conducted the formal assessments (27, 36, 39).

Case studies found inconclusive results related to the intervention’s efficacy. Most case studies reported a reduction after the intervention on outcomes such as intrusive thoughts (27), post-traumatic symptoms (28, 31, 38), anxiety (28, 33–35, 37–40), depression (28, 33, 34, 37–39), general clinical symptoms (30, 32), suicidal ideation (36) and insomnia (39). However, some studies did not indicate the cut-off selected to establish the recovery of symptoms and no size effect or significance of change was calculated. Consequently, out of 14 case studies, only 4 interventions based on CBT components as well as interpersonal relationships actually reported a total recovery of post-traumatic, anxiety and depressive symptoms (28, 33, 34, 38).

Along the same lines, controlled and non-controlled interventions showed a reduction in anxiety, depression, insomnia, stress, PTSD and COVID-19 fears (Table 2). However, a total recovery of symptoms was only reported in 13 out of the 29 studies. More precisely, a total recovery from anxiety symptoms was found in two studies (51, 53), recovery from COVID-19 fears was found in one study (42) and a total recovery from insomnia was found in one study (54). With regard to total recovery on multiple outcomes, complete disappearance of anxiety and depression was found in three studies (41, 50, 60). Two studies found a total recovery from symptoms of depression and PTSD (45) or depression and stress (52). Additionally, total recovery from three symptoms (anxiety, depression and insomnia/stress) was found in four studies (56, 57, 66, 67).

### 3.5 Risk of bias

As showed in Supplementary material D, the quality of case series studies was generally low (eight studies obtained 2 points out of 7 and two studies obtained 4 points). Only four studies could be classified as “good” (scores of 5–6 out of 9 points). For case studies rated as “poor,” the most important issues were related to the lack of information about the psychological intervention that was provided and not using valid and reliable measures. Other items that failed in almost all interventions were lack of follow-up assessments, not reporting statistical methods and poor results reports.

Supplementary material E shows the analyses of study quality for pre-post interventions without control group. All studies obtained scores from 4 to 7 points (out of 12 points) which may be interpreted as “fair” quality. The items that are more worrisome are item 4 (enrollment of potential participants), item 5 (sample size justification), item 6 (definition of the intervention), item 8 (blinded assessments), item 9 (dropouts) and item 11 (length of follow-up).

In third place, we analyzed randomized controlled trials. As shown in Supplementary material F, nine studies obtained scores of up to 7 points (out of 14) which could be interpreted as being “poor” quality studies. Another six studies were classified as “fair” studies because their total scores oscillated between 9 and 11 points. Just one study obtained 12 points, which indicated a “good” quality study. In general terms, RCT failed to provide proper information about participants and providers blinding to allocation, blinded assessments, adherence rates and pre-specified hypothesis.

The remaining five studies were non-randomized controlled interventions with control group. Although scores ranged from 6 to 8 points (out of 14), we considered that these interventions were of “good” quality because some items referred specifically to randomized studies (items 1–5). As these interventions were not described as randomized control trials, in most cases it was not possible to determine whether allocation and assessments were blinded. Additional shortcomings with these interventions were lack of information about adherence rates, sample size justification and lack of pre-specified hypothesis (Supplementary material G).

## 4 Discussion

Coping with the physical and social consequences of the pandemics was a great challenge for the entire population (71), and especially for those suffering from COVID-19 or long COVID-19 conditions (7, 12). This resulted in the emergence of psychological interventions to alleviate the psychological impact of the pandemic (18). The main aim of this systematic review was to summarize and analyze the psychological interventions that are available for patients suffering any kind of COVID-19 conditions and comorbid emotional disorders. This study provides results from 43 studies including 2,359 participants.

Due to the magnitude of the COVID-19 pandemic, several economic investments have been executed (72) specially in developed countries. However, as stated in previous lines, only 26% of studies included in this review have been developed in western countries. It seems that, despite the availability of economic resources and although we already have psychological interventions available to be provided for health conditions (73, 74), research efforts are not reaching the entire globe and psychological interventions are not yet equally distributed. We expect that future research will allow psychological interventions to be implemented in different countries and cultures and reach all COVID-19 patients who need it.

Another important finding from this systematic review is that almost all psychological interventions were provided to COVID-19 patients and only 3 studies were focused on post COVID-19 or long COVID-19 populations. This contrasts with the high prevalence of long COVID-19 syndrome and the negative consequences of not caring for this population. Scientific evidence highlights that around 10–20% of COVID-19 patients might develop long COVID-19 (75) and, what is more important, it seems that post COVID-19 patients are at risk of emotional suffering and suicide (76). Fortunately, it seems that programs addressing physical and psychological issues may reduce the emotional suffering and the risk of suicide in post COVID-19 patients (76). Taking this into account, future psychological interventions should specifically include post COVID-19 patients and analyze whether the same intervention could be applied to all COVID-19 patients irrespective of the duration of the COVID-19 symptoms.

With respect to COVID-19 severity, we found in our systematic review that investigations included very heterogeneous participants, from asymptomatic to patients with severe COVID-19 symptoms. It has been postulated that length of hospitalization and severity of COVID-19 symptoms are associated with reduced quality of life (77) so there is no doubt that, if possible, psychological interventions should be provided during and after discharge. However, patients with mild but chronic physical symptoms may also experience an impact on their quality of life, especially those with pulmonary affections (78) so we propose that all COVID-19 patients should be offered both preventive psychological interventions and psychological treatment. As length and duration of sessions was not clearly established across interventions, a stepped model of care (79, 80) could serve to determine the dosage, length and format of delivery for each patient.

In relation to the delivery format, the COVID-19 pandemic has evidenced that current mental health services are insufficient to care for all people who suffer emotional disorders and has provided an opportunity to implement new models of care (81). Furthermore, the mobility restrictions and lockdowns associated with the pandemic impeded the provision of face-to-face sessions, which also favored the development of new models of care. These facts were clearly observed in our systematic review by the great number of interventions that used technology both as the main format of delivery or as a complement to onsite sessions. We strongly believe that the use of audio-visual content, which is usually requested by patients and professionals (82), could be extremely beneficial for COVID-19 patients because they usually present with memory and attentional deficits (83). In this sense, technology-based psychological interventions help to provide audio-visual content that could be always accessible (84). It facilitates the access of participants to the intervention whenever they need it, patients are able to review and repeat the content, which may in turn result in higher skills acquisition (85). Another important outcome from our work is that different questionnaires were employed to assess emotional disorders in COVID-19 patients. Most of the studies used well established instruments designed for general populations (i.e., PHQ, BAI, GAD, SDS). Nonetheless, it has also been claimed there is a need to select the most appropriate questionnaire according to the specific circumstances of the participants who are being evaluated (86). Consequently, during the pandemic, enormous effort were carried out to develop COVID-19 specific measures (87). We need to consider that some physical symptoms of COVID-19 and long COVID-19 conditions include loss of attention, confusion, fatigue, difficulties in taking decisions or insomnia due to pain (88). These symptoms usually overlap with the main criteria used to diagnose anxiety and depressive symptoms (89). Future research should consider whether the use of general questionnaires may result in an over diagnosis of emotional disorders in COVID-19 populations and if we need to conduct separate and extensive assessments including cognitive-specific measures and psychological in-depth interviews.

The aforementioned assessments allow researchers to evaluate the efficacy of the interventions. Different therapies, such as CBT, interpersonal psychotherapy, positive psychology and mind–body approaches, have been proposed to address emotional disorders in COVID-19 patients. Our results indicated that, in general terms, a reduction in emotional disorders is found after psychological interventions. It is remarkable that RCTs based on CBT seem to be one of the most convenient interventions for the reduction of emotional suffering in COVID-19 patients, demonstrated by the efficacy rates and the low risk of bias of these studies. While acknowledging this valuable information, these results may be interpreted with caution as several limitations have been detected in this review. First, only 43 studies have been conducted since the onset of the pandemic, and few countries are represented in those studies, which may compromise generalization of findings. Second, most studies found only a partial recovery of symptoms and it is difficult to establish if emotional recovery is attributable to the psychological intervention itself or to a recovery from the COVID-19 physical symptoms. Third, there is a lack of well-designed and rigorous RCT and, as indicated by our risk of bias analyses, a worrisome percentage of studies did not provide enough information about the intervention that was provided, especially in case studies. Another shortcoming with psychological interventions is their insufficient length (sometimes programs were based on only one session), the lack of proper follow-up assessments (which may help to determine whether the improvement achieved disappeared with time or if the improvements were maintained), the inadequate assessment protocols and the lack of transdiagnostic approaches that allow to address the factors contributing to the development and maintenance of emotional disorders (90).

In this sense, it is remarkable that none of the aforementioned interventions proposed the implementation of a transdiagnostic psychological intervention (91). Given that comorbidity between anxiety and depression is highly frequent in COVID-19 patients (92), we postulate the need to develop and implement transdiagnostic CBT interventions. These interventions target etiological and maintenance factors shared by distinct emotional disorders (90) instead of focusing on specific symptoms (e.g., the Unified Protocol for transdiagnostic treatment of emotional disorders) (93). In recent years, different systematic reviews and meta-analysis have been published regarding their efficacy when applied to individuals with emotional disorders (94) and it has also been applied recently with encouraging results to individuals with comorbid emotional disorders and health conditions, including people with long COVID-19 (95). Transdiagnostic psychological treatments have multiple advantages, for example, clinicians can use one single treatment protocol for a variety of emotional disorders and comorbid cases, thus it is easier to train clinicians and to disseminate evidence-based psychological treatments (96). Finally, another advantage is the possibility to deliverer it in cost-effective formats such as group or technology-based interventions (97).

Arguments shown in this work may help to understand current practices in the context of COVID-19 patients and may help to expand the field of research. However, this work is not exempt from some limitations. First, systematic reviews usually present potential risk of bias (selection, attrition, interpretation of results etc.) (98). Although we have followed PRISMA recommendations, have pre-registered our work in PROSPERO and have included two independent researchers across all the process, it is possible that some biases are still present. Second, our objective was to summarize psychological interventions in the context of COVID-19 patients and we did not exclude any study due to their quality. As a result, some studies included in this review were rated as “poor” or “fair” quality. Related with this, although some studies included in this review (*n* = 9) administered pharmacotherapy (i.e., antidepressants), none of them conducted statistical analyses comparing participants which were taking pharmacotherapy with psychotherapy and those that received only psychotherapy. Thus we can not determine the independent percentage of change attributable to each of these two treatments (e.g., psychotherapy and pharmacotherapy). Future studies administering drugs should include formal analyses comparing populations with and without pharmacotherapy prescription to obtain more reliable results. Third, we have not included study protocols nor registers in clinicaltrials.gov as previous reviews did (99) so it is possible that some psychological interventions which are currently being implemented, especially in long COVID-19 patients, were not included in our review. Finally, due to the heterogeneity in the studies, it was not possible to conduct a meta-analysis which could facilitate generalization and comparison of results.

Despite these limitations, this systematic review could be useful both for researchers and clinical practice by providing an overview of current psychological interventions for COVID-19 patients. According to our results, future interventions should include long COVID-19 participants, offer preventive and treatment protocols to all COVID-19 patients, use more sophisticated research designs, propose transdiagnostic interventions with long-term follow-ups, explore which are the best assessment protocols and use cost-effective formats (i.e., group and self-administered interventions based on the use of technologies).

## Data availability statement

The original contributions presented in the study are included in the article/Supplementary material, further inquiries can be directed to the corresponding author.

## Author contributions

VM-B: Conceptualization, Data curation, Formal analysis, Investigation, Methodology, Software, Writing – original draft. LM-G: Data curation, Formal analysis, Investigation, Methodology, Software, Writing – original draft. ÓP-B: Investigation, Writing – original draft. JO: Conceptualization, Data curation, Funding acquisition, Methodology, Project administration, Supervision, Writing – review & editing. EC-B: Funding acquisition, Project administration, Supervision, Writing – review & editing.

## References

[1] World Health Orgnization [WHO]. WHO coronavirus disease (COVID-19) dashboard. WHO Heal Emerg Dashboard (2023)

[2] Chilamakuri R Agarwal S. COVID-19: characteristics and therapeutics. Cells. (2021) 10:206. doi: 10.3390/cells10020206, PMID: 33494237 PMC7909801

[3] al Maqbali M al badi K al Sinani M Madkhali N Dickens GL. Clinical features of COVID-19 patients in the first year of pandemic: a systematic review and Meta-analysis. Biol Res Nurs. (2022) 24:172–85. doi: 10.1177/10998004211055866, PMID: 34866409 PMC8968436

[4] World Health Organization [WHO]. WHO coronavirus (COVID-19) dashboard. *Glob Situat* (2023) Available at: https://covid19.who.int/?mapFilter=deaths (Accessed July 25, 2023)

[5] World Health Orgnization [WHO]. COVID-19: Clinical care. *A Clin case Defin post COVID-19 Cond by a Delphi consensus, 6 Oct 2021* (2021) Available at: https://www.who.int/publications/i/item/WHO-2019-nCoV-Post_COVID-19_condition-Clinical_case_definition-2021.1 (Accessed July 25, 2023)

[6] Mehandru S Merad M. Pathological sequelae of long-haul COVID. Nat Immunol. (2022) 23:194–202. doi: 10.1038/s41590-021-01104-y, PMID: 35105985 PMC9127978

[7] Vindegaard N Benros ME. COVID-19 pandemic and mental health consequences: systematic review of the current evidence. Brain Behav Immun. (2020) 89:531–42. doi: 10.1016/j.bbi.2020.05.048, PMID: 32485289 PMC7260522

[8] Tang F Liang J Zhang H Kelifa MM He Q Wang P. COVID-19 related depression and anxiety among quarantined respondents. Psychol Health. (2021) 36:164–78. doi: 10.1080/08870446.2020.1782410 32567952

[9] Ubillos-Landa S Puente-Martínez A González-Castro JL. Psychological withdrawal and mental health during the COVID-19 pandemic: a longitudinal study. Psychol Health. (2023) 38:1361–77. doi: 10.1080/08870446.2021.2019254 34955057

[10] Morris SG Kudrna L Martin J. Mental health and life satisfaction among those advised to shield during the COVID-19 pandemic in the UK: a secondary analysis of the understanding society longitudinal study. Front Public Health. (2023) 11:1235903. doi: 10.3389/fpubh.2023.1235903, PMID: 37829093 PMC10566375

[11] Barbalat G Tanguy Melac A Zante E Haesebaert F Franck N. Predictors of mental well-being over the first lockdown period due to the COVID-19 pandemic in France. A repeated cross-sectional study. Front Public Health. (2023) 11:1234023. doi: 10.3389/fpubh.2023.1234023, PMID: 37701911 PMC10493269

[12] Khraisat B Toubasi A AlZoubi L Al-Sayegh T Mansour A. Meta-analysis of prevalence: the psychological sequelae among COVID-19 survivors. Int J Psychiatry Clin Pract. (2022) 26:234–43. doi: 10.1080/13651501.2021.1993924, PMID: 34709105

[13] Amin S Mehmood W Aman-Ullah A Khan MA. Corona-phobia violated human rights? Impact of COVID-19 on patient’s well-being. Int J Hum Rights Healthc. (2022). doi: 10.1108/IJHRH-05-2022-0048 [Epubh ahead of print].

[14] O’Brien H Tracey MJ Ottewill C O’Brien ME Morgan RK Costello RW . An integrated multidisciplinary model of COVID-19 recovery care. Ir J Med Sci. (2021) 190:461–8. doi: 10.1007/s11845-020-02354-9, PMID: 32894436 PMC7475726

[15] Wen W Chen C Tang J Wang C Zhou M Cheng Y . Efficacy and safety of three new oral antiviral treatment (molnupiravir, fluvoxamine and Paxlovid) for COVID-19:a meta-analysis. Ann Med. (2022) 54:516–23. doi: 10.1080/07853890.2022.2034936, PMID: 35118917 PMC8820829

[16] Xavier-Santos D Padilha M Fabiano GA Vinderola G Gomes Cruz A Sivieri K . Evidences and perspectives of the use of probiotics, prebiotics, synbiotics, and postbiotics as adjuvants for prevention and treatment of COVID-19: a bibliometric analysis and systematic review. Trends Food Sci Technol. (2022) 120:174–92. doi: 10.1016/j.tifs.2021.12.033, PMID: 35002079 PMC8720301

[17] Décary S De Groote W Arienti C Kiekens C Boldrini P Giuseppe Lazzarini S . Scoping review of rehabilitation care models for post COVID-19 condition. Bull World Health Organ. (2022) 100:676–88. doi: 10.2471/BLT.22.288105, PMID: 36324552 PMC9589389

[18] Rahmati F Khalili R. Investigating the effectiveness of psychological interventions in response to stress, anxiety, and depression in coronavirus disease 2019 patients: a systematic review. J Educ Health Promot. (2022) 11:203–6. doi: 10.4103/jehp.jehp_923_21 36003242 PMC9393944

[19] Tasleem A Wang Y Li K Jiang X Krishnan A He C . Effects of mental health interventions among people hospitalized with COVID-19 infection: a systematic review of randomized controlled trials. Gen Hosp Psychiatry. (2022) 77:40–68. doi: 10.1016/j.genhosppsych.2022.04.002, PMID: 35533528 PMC8993417

[20] Komariah M Amirah S Faisal EG Prayogo SA Maulana S Platini H . Efficacy of internet-based cognitive behavioral therapy for depression and anxiety among global population during the COVID-19 pandemic: a systematic review and meta-analysis of a randomized controlled trial study. Healthcare. (2022) 10:1224. doi: 10.3390/healthcare10071224, PMID: 35885751 PMC9315502

[21] Chennapragada L Sullivan SR Hamerling-Potts KK Tran H Szeszko J Wrobleski J . International PRISMA scoping review to understand mental health interventions for depression in COVID-19 patients. Psychiatry Res. (2022) 316:114748. doi: 10.1016/j.psychres.2022.114748, PMID: 35944370 PMC9313534

[22] Safieh J Broughan J McCombe G McCarthy N Frawley T Guerandel A . Interventions to optimise mental health outcomes during the COVID-19 pandemic: a scoping review. Int J Ment Health Addict. (2022) 20:2934–55. doi: 10.1007/s11469-021-00558-3, PMID: 34149329 PMC8204922

[23] Page MJ McKenzie JE Bossuyt PM Boutron I Hoffmann TC Mulrow CD . The PRISMA 2020 statement: an updated guideline for reporting systematic reviews. PLoS Med. (2021) 18:e1003583. doi: 10.1371/journal.pmed.1003583, PMID: 33780438 PMC8007028

[24] Mendeley Ltd. Mendeley Desktop. (2020)

[25] Ouzzani M Hammady H Fedorowicz Z Elmagarmid A. Rayyan – a web and mobile app for systematic reviews. Syst Rev. (2016) 5:210. doi: 10.1186/s13643-016-0384-4, PMID: 27919275 PMC5139140

[26] Li T Higgins J Deeks J. Collecting data In: Higgins J Thomas J Chandler J Cumpston M Li T Page M Welch V, editors. Cochrane handbook for systematic reviews of interventions version 6.3. London: Cochrane (2022)

[27] Alkhamees AA. Obsessive–compulsive disorder post-COVID-19: A case presentation. Egypt J Neurol Psychiatr Neurosurg. (2021) 57:1–3. doi: 10.1186/s41983-021-00405-1 PMC8557101 34744416

[28] Bogucki OE Sawchuk CN. Cognitive processing therapy for posttraumatic stress disorder due to COVID-19-related traumas: a case study. Psychol Serv. (2022) 20:533–7. doi: 10.1037/ser0000630, PMID: 35113624

[29] Callus E. Provision of psychological support to a 31-year-old man with SARS-CoV2-induced pneumonia during and after hospitalization: a clinical case report. Int J Environ Res Public Health. (2023) 20:757. doi: 10.3390/ijerph20010757, PMID: 36613078 PMC9819220

[30] Chen J Tian C Cheng X Huang Y Tang L Wang R . A case of coronvirus disease 2019 with psychological disorders. Psychiatr Danub. (2020) 32:581–3. doi: 10.24869/psyd.2020.581 33370770

[31] Dinapoli L Ferrarese D Belella D Carnevale S Camardese G Sani G . Psychological treatment of traumatic memories in COVID-19 survivors. Clin Psychol Psychother. (2023) 30:225–33. doi: 10.1002/cpp.2771, PMID: 35916065

[32] Edet BE Essien EA Ugobo MB Okafor CJ Olose EO Essien VA. Depression and suicidality in a COVID-19 patient: a case report from Calabar, Nigeria. West Afr J Med. (2022) 39:548–51. PMID: 35633651

[33] Hu C-C Huang J-W Wei N Hu S-H Hu J-B Li S-G . Interpersonal psychotherapy-based psychological intervention for patient suffering from COVID-19: a case report. World J Clin Cases. (2020) 8:6064–70. doi: 10.12998/wjcc.v8.i23.6064, PMID: 33344606 PMC7723699

[34] Huang J-W Zhou X-Y Lu S-J Xu Y Hu J-B Huang M-L . Dialectical behavior therapy-based psychological intervention for woman in late pregnancy and early postpartum suffering from COVID-19: a case report. J Zhejiang Univ Sci B. (2020) 21:394–9. doi: 10.1631/jzus.B2010012, PMID: 32425005 PMC7110264

[35] Khawam E Khouli H Pozuelo L. Treating acute anxiety in patients with COVID-19. Cleve Clin J Med. (2020). doi: 10.3949/ccjm.87a.ccc016 [Epubh ahead of print]. 32409438

[36] Naskar C Grover S Sahoo S Mehra A. Managing a COVID-positive health-care worker with recent suicide attempt through telepsychiatry. Ann Indian Psychiatry. (2022) 6:99–101. doi: 10.4103/aip.aip_4_21

[37] Nuertey BD Mumuni K Addai J Kunfah S Attibu RI Acquah D . Attempted suicide of two confirmed SARS-CoV-2 infected patients in an isolation facility and recommendations to prevent COVID-19 suicides: a case report. Pan Afr Med J. (2022) 41:245. doi: 10.11604/pamj.2022.41.245.29660, PMID: 35734314 PMC9187986

[38] Sadeghi M Moradi Z Ershadi F. The clinical trial of COVID-19 patients: the effectiveness of emotion-focused therapy on post-traumatic stress and depression. Iran Red Crescent Med J. (2021) 23:e998. doi: 10.32592/ircmj.2021.23.10.998

[39] Situmorang DDB. “When the first session may be the last!”: a case report of the implementation of “rapid tele-psychotherapy” with single-session music therapy in the COVID-19 outbreak. Palliat Support Care. (2022) 20:290–5. doi: 10.1017/S1478951521001425, PMID: 34399867 PMC8458841

[40] Taube M. Depression and brain fog as long-COVID mental health consequences: difficult, complex and partially successful treatment of a 72-year-old patient—a case report. Front Psychiatry. (2023) 14:1153512. doi: 10.3389/fpsyt.2023.1153512, PMID: 37032935 PMC10079873

[41] Biagianti B Lisi I Di Liberto A Turtulici N Foti G Zito S . Feasibility and preliminary efficacy of brief tele-psychotherapy for COVID-19 patients and their first-degree relatives. J Affect Disord. (2023) 330:300–8. doi: 10.1016/j.jad.2023.03.024, PMID: 36934855 PMC10022466

[42] Brennstuhl M-J Pascale T Ann RJ Louise TC Lydia P Christine R . Treating COVID-19 patients with EMDR: a pilot study. Eur J Trauma Dissociation. (2022) 6:100276. doi: 10.1016/j.ejtd.2022.100276, PMID: 37521717 PMC9065594

[43] Öner Cengiz H Ayhan M Güner R. Effect of deep breathing exercise with Triflo on dyspnoea, anxiety and quality of life in patients receiving covid-19 treatment: a randomized controlled trial. J Clin Nurs. (2021) 31:3439–53. doi: 10.1111/jocn.16171, PMID: 34897869

[44] Compagno S Palermi S Pescatore V Brugin E Sarto M Marin R . Physical and psychological reconditioning in long COVID syndrome: Results of an out-of-hospital exercise and psychological-based rehabilitation program. Int J Cardiol Heart Vasc. (2022) 41:101080. doi: 10.1016/j.ijcha.2022.101080 35854691 PMC9286763

[45] Fan Y Shi Y Zhang J Sun D Wang X Fu G . The effects of narrative exposure therapy on COVID-19 patients with post-traumatic stress symptoms: a randomized controlled trial. J Affect Disord. (2021) 293:141–7. doi: 10.1016/j.jad.2021.06.019, PMID: 34186232 PMC8234566

[46] Ganesan S Balasubramanian B Krishnamurthy P Govindan R Mani N. Effects of tele-counseling on reducing anxiety levels of COVID-19 patients in isolation wards: an observational study. Indian J Psychol Med. (2023) 45:43–6. doi: 10.1177/02537176221139598, PMID: 36778622 PMC9896101

[47] Torbati AG Imeni M Abbaspour S. Impact of dialectical behavior therapy on depression and anxiety in patients following COVID-19 discharge. Open Psychol J. (2022) 16:1–7. doi: 10.2174/18743501-v16-e2208191

[48] Ghodrati-Torbati AG Abbaspour S Zandi A. Efficacy of psychoeducational intervention on depression and anxiety after discharge in patients with covid-19. J Public Health Dev. (2022) 20:209–20. doi: 10.55131/jphd/2022/200317

[49] Kim J-W Stewart R Kang S-J Jung S-I Kim S-W Kim J-M. Telephone based interventions for psychological problems in hospital isolated patients with COVID-19. Clin Psychopharmacol Neurosci. (2020) 18:616–20. doi: 10.9758/cpn.2020.18.4.616, PMID: 33124594 PMC7609215

[50] Kong X Kong F Zheng K Tang M Chen Y Zhou J . Effect of psychological–behavioral intervention on the depression and anxiety of COVID-19 patients. Front Psychiatry. (2020) 11:586355. doi: 10.3389/fpsyt.2020.586355 33329130 PMC7715028

[51] Lerthattasilp T Kosulwit L Phanasathit M Nuallaong W Tapanadechopone P Thanetnit C . Effect of an online psychological support group on patients with COVID-19 in a Thai field hospital: a real world study. J Health Res. (2021) 36:1040–6. doi: 10.1108/JHR-01-2021-0044

[52] Li J Li X Jiang J Xu X Wu J Xu Y . The effect of cognitive behavioral therapy on depression, anxiety, and stress in patients with COVID-19: a randomized controlled trial. Front Psychiatry. (2020) 11:580827. doi: 10.3389/fpsyt.2020.580827, PMID: 33192723 PMC7661854

[53] Liu K Chen Y Wu D Lin R Wang Z Pan L. Effects of progressive muscle relaxation on anxiety and sleep quality in patients with COVID-19. Complement Ther Clin Pract. (2020) 39:101132. doi: 10.1016/j.ctcp.2020.101132, PMID: 32379667 PMC7102525

[54] Liu Y Yang Y-Q Liu YY Pei S-L Yang H-H Wu J-J . Effects of group psychological intervention combined with pulmonary rehabilitation exercises on anxiety and sleep disorders in patients with mild coronavirus disease 2019 (COVID-19) infections in a Fangcang hospital. Psychol Health Med. (2021) 27:333–42. doi: 10.1080/13548506.2021.1916956, PMID: 33877926

[55] Liu Z Qiao D Xu Y Zhao W Yang Y Wen D . The efficacy of computerized cognitive behavioral therapy for depressive and anxiety symptoms in patients with COVID-19: randomized controlled trial. J Med Internet Res. (2021) 23:e26883. doi: 10.2196/26883, PMID: 33900931 PMC8128049

[56] Mahendru K Pandit A Singh V Choudhary N Mohan A Bhatnagar S. Effect of meditation and breathing exercises on the well-being of patients with SARS-CoV-2 infection under institutional isolation: a randomized control trial. Indian J Palliat Care. (2021) 27:490–4. doi: 10.25259/IJPC_40_21 34898943 PMC8655642

[57] Maresca G Formica C De Cola MC Lo Buono V Latella D Cimino V . Care models for mental health in a population of patients affected by COVID-19. J Int Med Res. (2022) 50:030006052210974. doi: 10.1177/03000605221097478, PMID: 35531918 PMC9092593

[58] Parizad N Goli R Faraji N Mam-Qaderi M Mirzaee R Gharebaghi N . Effect of guided imagery on anxiety, muscle pain, and vital signs in patients with COVID-19: a randomized controlled trial. Complement Ther Clin Pract. (2021) 43:101335. doi: 10.1016/j.ctcp.2021.101335, PMID: 33647676 PMC7982304

[59] Priyamvada R Ranjan R Chaudhury S. Efficacy of psychological intervention in patients with post-COVID-19 anxiety. Ind Psychiatry J. (2021) 30:41–S44. doi: 10.4103/0972-6748.328787, PMID: 34908663 PMC8611569

[60] Rutkowski S Bogacz K Czech O Rutkowska A Szczegielniak J. Effectiveness of an inpatient virtual reality-based pulmonary rehabilitation program among COVID-19 patients on symptoms of anxiety, depression and quality of life: preliminary results from a randomized controlled trial. Int J Environ Res Public Health. (2022) 19:16980. doi: 10.3390/ijerph192416980, PMID: 36554860 PMC9779397

[61] Shaygan M Yazdani Z Rambod M. The effect of interactive virtual psycho-educational interventions via social networks on self-efficacy and anxiety among patients infected with COVID-19 and living in home quarantine: a randomized control trial. Iran J Nurs Midwifery Res. (2023) 28:65–71. doi: 10.4103/ijnmr.ijnmr_451_21 37250934 PMC10215544

[62] Sotoudeh HG Alavi SS Akbari Z Jannatifard F Artounian V. The effect of brief crisis intervention package on improving quality of life and mental health in patients with COVID-19. Iran J Psychiatry. (2020) 15:205–12. doi: 10.18502/ijps.v15i3.3812, PMID: 33193768 PMC7603589

[63] Sun P Fan D-J He T Li H-Z Wang G Zhang X-Z . The effects of psychological intervention on anxiety symptoms of COVID19-positive patients isolated in hospital wards. Eur Rev Med Pharmacol Sci. (2021) 25:498–502. doi: 10.26355/eurrev_202101_24421, PMID: 33506941

[64] Wei N Huang B Lu SJ Hu JB Zhou XY Hu CC . Efficacy of internet-based integrated intervention on depression and anxiety symptoms in patients with COVID-19. J Zhejiang Univ Sci B. (2020) 21:400–4. doi: 10.1631/jzus.B2010013, PMID: 32425006 PMC7203540

[65] Won G Lee HJ Lee JH Choi TY Hong H-L Jung CY. Impact of a psychiatric consultation program on COVID-19 patients: an experimental study. Psychiatry Investig. (2023) 20:471–80. doi: 10.30773/pi.2022.0295, PMID: 37253473 PMC10232054

[66] Xiao CX Lin YJ Lin RQ Liu AN Zhong GQ Lan CF. Effects of progressive muscle relaxation training on negative emotions and sleep quality in COVID-19 patients: a clinical observational study. Medicine (Baltimore). (2020) 99:e23185. doi: 10.1097/MD.0000000000023185 33217826 PMC7676563

[67] Yang X Yang X Kumar P Cao B Ma X Li T. Social support and clinical improvement in COVID-19 positive patients in China. Nurs Outlook. (2020) 68:830–7. doi: 10.1016/j.outlook.2020.08.008, PMID: 32980152 PMC7444976

[68] Yuan L-P Yu Z-H Zhang X-C Zhang W Jin L-L Wang Z . The psychological effect of forming WeChat groups between medical staff and patients with COVID-19. Front Public Health. (2021) 9:586465. doi: 10.3389/fpubh.2021.586465, PMID: 34249824 PMC8260973

[69] Zheng T Lin J Tu L Hu J Wei W. Correlation analysis of positive therapy based on high content image analysis technology on posttraumatic nerve growth in patients with COVID-19 in the context of intelligent medical treatment. Contrast Media Mol Imaging. (2022) 2022:9165764. doi: 10.1155/2022/9165764 35935332 PMC9297126

[70] National Heart Lung and Blood Institute [NHLBI]. Study quality assessment tools. (2021) Available at: https://www.nhlbi.nih.gov/health-topics/study-quality-assessment-tools (Accessed March 25, 2020)

[71] Lin C-C Yeh C-B. Psychological impacts of COVID-19 pandemic. J Med Sci. (2023) 43:1. doi: 10.4103/jmedsci.jmedsci_269_21

[72] European Commission. Cohesion policy action against coronavirus. *Reg Policy Funding* (2023) Available at: https://ec.europa.eu/regional_policy/funding/coronavirus-response_en (Accessed July 28, 2023).

[73] Thompson RD Delaney P Flores I Szigethy E. Cognitive-behavioral therapy for children with comorbid physical illness. Child Adolesc Psychiatr Clin N Am. (2011) 20:329–48. doi: 10.1016/j.chc.2011.01.013 21440859

[74] McCombie A Gearry R Andrews J Mikocka-Walus A Mulder R. Computerised cognitive behavioural therapy for psychological distress in patients with physical illnesses: a systematic review. J Clin Psychol Med Settings. (2015) 22:20–44. doi: 10.1007/s10880-015-9420-0, PMID: 25666485

[75] World Health Organization [WHO]. Post COVID-19 condition (long COVID). (2022) Available at: https://www.who.int/europe/news-room/fact-sheets/item/post-covid-19-condition (Accessed January 9, 2023)

[76] Simonetti A Bernardi E Janiri D Mazza M Montanari S Catinari A . Suicide risk in post-COVID-19 syndrome. J Pers Med. (2022) 12:2019. doi: 10.3390/jpm12122019, PMID: 36556240 PMC9785632

[77] Kaso AW Tesema HG Hareru HE Kaso T Ashuro Z Talemahu AA . Health-related quality of life and associated factors among Covid-19 survivors. Experience from Ethiopian treatment centers. Infect Drug Resist. (2022) 15:6143–53. doi: 10.2147/IDR.S386566, PMID: 36304968 PMC9593469

[78] Siboni F Alimoradi Z Atashi V Alipour M Khatooni M. Quality of life in different chronic diseases and its related factors. Int J Prev Med. (2019) 10:65. doi: 10.4103/ijpvm.IJPVM_429_17, PMID: 31198500 PMC6547796

[79] Bower P Gilbody S. Stepped care in psychological therapies: access, effectiveness and efficiency. Br J Psychiatry. (2005) 186:11–7. doi: 10.1192/bjp.186.1.11, PMID: 15630118

[80] Cross S Hickie I. Transdiagnostic stepped care in mental health. Public Health Res Pract. (2017) 27:27. doi: 10.17061/phrp2721712 28474049

[81] Yang X. The impact of COVID-19 on access to mental health services and the use of teletherapy as an alternative form of treatment. Arch Clin Psychiatry. (2022) 49:23–30. doi: 10.15761/0101-60830000000423

[82] Osma J Martínez-García L Prado-Abril J Peris-Baquero Ó González-Pérez A. Developing a smartphone app based on the unified protocol for the transdiagnostic treatment of emotional disorders: a qualitative analysis of users and professionals’ perspectives. Internet Interv. (2022) 30:100577–7. doi: 10.1016/j.invent.2022.100577, PMID: 36213084 PMC9535424

[83] Velichkovsky BB Razvaliaeva AY Khlebnikova AA Manukyan PA Kasatkin VN. Attention and memory after COVID-19 as measured by neuropsychological tests: systematic review and meta-analysis. Acta Psychol. (2023) 233:103838. doi: 10.1016/j.actpsy.2023.103838, PMID: 36657196 PMC9834202

[84] Stoll J Müller JA Trachsel M. Ethical issues in online psychotherapy: a narrative review. Front Psychiatry. (2020) 10:993. doi: 10.3389/fpsyt.2019.00993, PMID: 32116819 PMC7026245

[85] Hardenberg J Rana I Tori K. Evaluating impact of repeated exposure to high Fidelity simulation: skills acquisition and stress levels in postgraduate critical care nursing students. Clin Simul Nurs. (2020) 48:96–102. doi: 10.1016/j.ecns.2020.06.002

[86] Institute of Medicine. Overview of psychological testing In: Psychological testing in the Service of Disability Determination. Committee on Psychological Testing, Including Validity Testing, for Social Security Administration Disability Determinations. Washington (DC): National Academies Press (US) (2015). doi: 10.17226/21704 26203491

[87] Cortez PA Joseph SJ Das N Bhandari SS Shoib S. Tools to measure the psychological impact of the COVID-19 pandemic: what do we have in the platter? Asian J Psychiatr. (2020) 53:102371. doi: 10.1016/j.ajp.2020.102371, PMID: 32891929 PMC7456260

[88] Farooqi M Khan A Jacobs A D’Souza V Consiglio F Karmen CL . Examining the long-term sequelae of SARS-CoV2 infection in patients seen in an outpatient psychiatric department. Neuropsychiatr Dis Treat. (2022) 18:1259–68. doi: 10.2147/NDT.S357262, PMID: 35761861 PMC9233564

[89] American Psychiatric Association. Diagnostic and statistical manual of mental disorders (DSM-5). Washington, D.C: American Psychiatric Press (2013).

[90] Barlow D Allen L Choate M. Toward a unified treatment for emotional disorders. Behav Ther. (2004) 35:205–30. doi: 10.1016/S0005-7894(04)80036-4 27993336

[91] Osma J Martínez-García L Quilez-Orden A Peris-Baquero Ó. Unified protocol for the transdiagnostic treatment of emotional disorders in medical conditions: a systematic review. Int J Environ Res Public Health. (2021) 18:5077. doi: 10.3390/ijerph18105077 34064898 PMC8151777

[92] Klaser K Hompson E Nguyen L Sudre C Al E. Anxiety and depression symptoms after COVID-19 infection: results from the COVID symptom study app. J Neurol Neurosurg Psychiatry. (2021) 92:1254–8. doi: 10.1136/jnnp-2021-327565, PMID: 34583944 PMC8599635

[93] Barlow D Farchione T Sauer-Zavala S Latin H Ellard K Bullis J . Unified protocol for transdiagnostic treatment of emotional disorders: therapist guide. 2nd ed. New York, NY: Oxford University Press (2018).

[94] Cassiello-Robbins CS Southward MW Tirpak JW Sauer-Zavala S. A systematic review of unified protocol applications with adult populations: facilitating widespread dissemination via adaptability. Clin Psychol Rev. (2020) 78:101852. doi: 10.1016/j.cpr.2020.101852, PMID: 32360953

[95] Martínez-Borba V Peris-Baquero O Martínez-García L Osma J del Corral BE. Unified protocol application in patients with long COVID-19 conditions In: Osma J Farchione T, editors. Applications of the unified protocol in health conditions. Oxford: Oxford University Press (2023)

[96] Cassiello-Robbins C Murray-Latin H Sauer-Zavala S. The unified protocol: future directions In: Barlow D Farchione T, editors. Applications of the unified protocol for transdiagnostic treatment of emotional disorders. Oxford: Oxford University Press (2018). 291–301.

[97] Peris-Baquero Ó Moreno JD Osma J. Long-term cost-effectiveness of group unified protocol in the Spanish public mental health system. Curr Psychol. (2022) 42:22462–77. doi: 10.1007/s12144-022-03365-8

[98] Owens JK. Systematic reviews: brief overview of methods, limitations, and resources. Nurse Author Ed. (2021) 31:69–72. doi: 10.1111/nae2.28

[99] Hawke LD Nguyen ATP Ski CF Thompson DR Ma C Castle D. Interventions for mental health, cognition, and psychological wellbeing in long COVID: a systematic review of registered trials. Psychol Med. (2022) 52:2426–40. doi: 10.1017/S0033291722002203, PMID: 35768406 PMC9300978

