# Supplementary material for: Guiding future research on psychological interventions in people with COVID-19 and post COVID syndrome and comorbid emotional disorders based on a systematic review
Source: Front Public Health. 2024 Jan 11;11:1305463. doi: 10.3389/fpubh.2023.1305463 (PMC10808326; doi:10.3389/fpubh.2023.1305463)
Supplement: Supplementary file 1 [file Table_1.docx]

Supplementary Material

Guiding future research on psychological interventions in people with COVID-19 and post COVID syndrome and comorbid emotional disorders based on a systematic review

Verónica Martínez-Borba^1,2^, Laura Martínez-García^1,2^, Óscar Peris-Baquero^1,2^, Jorge Osma^1,2*^, Esther del Corral-Beamonte^3^

*** Correspondence:** Jorge Osma: [osma@unizar.es](mailto:osma@unizar.es)

# Supplementary Data

**Supplementary material A.** PRISMA checklist for systematic reviews.

| **Section / Topic** | **Item #** | **Checklist item** | **Location** |
| --- | --- | --- | --- |
| **TITLE** | | |  |
| Title | 1 | Identify the report as a systematic review. | Page 1 |
| **ABSTRACT** | | |  |
| Abstract | 2 | See the PRISMA 2020 for Abstracts checklist. | Suppl.material B |
| **INTRODUCTION** | | |  |
| Rationale | 3 | Describe the rationale for the review in the context of existing knowledge. | Page 2 |
| Objectives | 4 | Provide an explicit statement of the objective(s) or question(s) the review addresses. | Page 2 |
| **METHODS** | | |  |
| Eligibility criteria | 5 | Specify the inclusion and exclusion criteria for the review and how studies were grouped for the syntheses. | Page 2 |
| Information sources | 6 | Specify all databases, registers, websites, organisations, reference lists and other sources searched or consulted to identify studies. Specify the date when each source was last searched or consulted. | Page 3 |
| Search strategy | 7 | Present the full search strategies for all databases, registers and websites, including any filters and limits used. | Suppl. material C |
| Selection process | 8 | Specify the methods used to decide whether a study met the inclusion criteria of the review, including how many reviewers screened each record and each report retrieved, whether they worked independently, and if applicable, details of automation tools used in the process. | Page 3 |
| Data collection process | 9 | Specify the methods used to collect data from reports, including how many reviewers collected data from each report, whether they worked independently, any processes for obtaining or confirming data from study investigators, and if applicable, details of automation tools used in the process. | Page 3 |
| Data items | 10a | List and define all outcomes for which data were sought. Specify whether all results that were compatible with each outcome domain in each study were sought (e.g. for all measures, time points, analyses), and if not, the methods used to decide which results to collect. | Page 3 |
|  | 10b | List and define all other variables for which data were sought (e.g. participant and intervention characteristics, funding sources). Describe any assumptions made about any missing or unclear information. | Page 3 |
| Study risk of bias assessment | 11 | Specify the methods used to assess risk of bias in the included studies, including details of the tool(s) used, how many reviewers assessed each study and whether they worked independently, and if applicable, details of automation tools used in the process. | Page 3 |
| Effect measures | 12 | Specify for each outcome the effect measure(s) (e.g. risk ratio, mean difference) used in the synthesis or presentation of results. | Page 3 |
| Synthesis methods | 13a | Describe the processes used to decide which studies were eligible for each synthesis [e.g. tabulating the study intervention characteristics and comparing against the planned groups for each synthesis (item #5)]. | Page 3 |
|  | 13b | Describe any methods required to prepare the data for presentation or synthesis, such as handling of missing summary statistics, or data conversions. | Page 3 |
|  | 13c | Describe any methods used to tabulate or visually display results of individual studies and syntheses. | Page 3 |
|  | 13d | Describe any methods used to synthesize results and provide a rationale for the choice(s). If meta-analysis was performed, describe the model(s), method(s) to identify the presence and extent of statistical heterogeneity, and software package(s) used. | Page 3 |
|  | 13e | Describe any methods used to explore possible causes of heterogeneity among study results (e.g. subgroup analysis, meta-regression). | Pages 3, 20 |
|  | 13f | Describe any sensitivity analyses conducted to assess robustness of the synthesized results. | Pages 3, 20 |
| Reporting bias assessment | 14 | Describe any methods used to assess risk of bias due to missing results in a synthesis (arising from reporting biases). | Page 3 |
| Certainty assessment | 15 | Describe any methods used to assess certainty (or confidence) in the body of evidence for an outcome. | Page 3 |
| **RESULTS** | | |  |
| Study selection | 16a | Describe the results of the search and selection process, from the number of records identified in the search to the number of studies included in the review, ideally using a flow diagram. | Figure 1, page 3 |
|  | 16b | Cite studies that might appear to meet the inclusion criteria, but which were excluded, and explain why they were excluded. | Figure 1 |
| Study characteristics | 17 | Cite each included study and present its characteristics. | Tables 1, 2 |
| Risk of bias in studies | 18 | Present assessments of risk of bias for each included study. | Suppl. materials D-G |
| Results of individual studies | 19 | For all outcomes, present, for each study: (a) summary statistics for each group (where appropriate) and (b) an effect estimate and its precision (e.g. confidence/credible interval), ideally using structured tables or plots. | Tables 1 and 2 |
| Results of syntheses | 20a | For each synthesis, briefly summarise the characteristics and risk of bias among contributing studies. | Pages 3, 17-19 |
|  | 20b | Present results of all statistical syntheses conducted. If meta-analysis was done, present for each the summary estimate and its precision (e.g. confidence/credible interval) and measures of statistical heterogeneity. If comparing groups, describe the direction of the effect. | Table 2, Pages 3, 17-19 |
|  | 20c | Present results of all investigations of possible causes of heterogeneity among study results. | Pages 19-21 |
|  | 20d | Present results of all sensitivity analyses conducted to assess the robustness of the synthesized results. | Pages 3, 17-19 |
| Reporting biases | 21 | Present assessments of risk of bias due to missing results (arising from reporting biases) for each synthesis assessed. | Pages 18-19 |
| Certainty of evidence | 22 | Present assessments of certainty (or confidence) in the body of evidence for each outcome assessed. | Pages 18-19 |
| **DISCUSSION** | | |  |
| Discussion | 23a | Provide a general interpretation of the results in the context of other evidence. | Pages 19-21 |
|  | 23b | Discuss any limitations of the evidence included in the review. | Page 20 |
|  | 23c | Discuss any limitations of the review processes used. | Page 20 |
|  | 23d | Discuss implications of the results for practice, policy, and future research. | Pages 19-21 |
| **OTHER INFORMATION** | | |  |
| Registration and protocol | 24a | Provide registration information for the review, including register name and registration number, or state that the review was not registered. | Page 1 |
|  | 24b | Indicate where the review protocol can be accessed, or state that a protocol was not prepared. | Page 1 |
|  | 24c | Describe and explain any amendments to information provided at registration or in the protocol. | Page 3 |
| Support | 25 | Describe sources of financial or non-financial support for the review, and the role of the funders or sponsors in the review. | Page 21 |
| Competing interests | 26 | Declare any competing interests of review authors. | Page 21 |
| Availability of data, code and other materials | 27 | Report which of the following are publicly available and where they can be found: template data collection forms; data extracted from included studies; data used for all analyses; analytic code; any other materials used in the review. | Page 21 |

**Supplementary material B**. PRISMA checklist for systematic reviews (abstract version).

| **Section and Topic** | **Item #** | **Checklist item** | **Reported (Yes/No)** |
| --- | --- | --- | --- |
| **TITLE** | | |  |
| Title | 1 | Identify the report as a systematic review. | Yes |
| **BACKGROUND** | | |  |
| Objectives | 2 | Provide an explicit statement of the main objective(s) or question(s) the review addresses. | Yes |
| **METHODS** | | |  |
| Eligibility criteria | 3 | Specify the inclusion and exclusion criteria for the review. | No (word limit) |
| Information sources | 4 | Specify the information sources (e.g. databases, registers) used to identify studies and the date when each was last searched. | Yes |
| Risk of bias | 5 | Specify the methods used to assess risk of bias in the included studies. | Yes |
| Synthesis of results | 6 | Specify the methods used to present and synthesise results. | No (word limit) |
| **RESULTS** | | |  |
| Included studies | 7 | Give the total number of included studies and participants and summarise relevant characteristics of studies. | Yes |
| Synthesis of results | 8 | Present results for main outcomes, preferably indicating the number of included studies and participants for each. If meta-analysis was done, report the summary estimate and confidence/credible interval. If comparing groups, indicate the direction of the effect (i.e. which group is favoured). | Yes |
| **DISCUSSION** | | |  |
| Limitations of evidence | 9 | Provide a brief summary of the limitations of the evidence included in the review (e.g. study risk of bias, inconsistency and imprecision). | Yes |
| Interpretation | 10 | Provide a general interpretation of the results and important implications. | Yes |
| **OTHER** | | |  |
| Funding | 11 | Specify the primary source of funding for the review. | No (word limit) |
| Registration | 12 | Provide the register name and registration number. | Yes |

**Supplementary material C**. Detailed searches for each database.

The same combination of words was used for all databases. Three domains (COVID-19 conditions, psychological interventions and psychological issues) were introduced and combined throughout Booleans operators. Words within a domain were separated by OR while words between domains were separated with AND.

Psychological intervention

Psychological issue

COVID-19

COVID-19 OR coronavirus OR “SARS CoV-2” OR “persistent COVID-19” OR long-COVID OR post-COVID OR “chronic COVID-19”

AND

“psychological intervention” OR “psychological treatment” OR “psychological program” OR psychotherapy

AND

“affective disorder” OR “mood disorder” OR “emotional disorder” OR anxiety OR depressive OR depression OR PTSD OR “posttraumatic stress” OR “post-traumatic stress” OR OCD OR obsessive-compulsive OR “obsessive compulsive” OR phobia OR panic OR agoraphobia OR dysthymia OR dysthymic OR hypochondria OR “social anxiety”

Keywords were searched preferably on title, abstract and keywords fields. However, not all databases allow this refining criteria. As different databases provide specific field options, we report the field selected in each database:

| **Database** | **Field** |
| --- | --- |
| Web of science | Topic (title, abstract and indexing) |
| Pubmed | Title/Abstract |
| Scopus | Article title, Abstract, Keywords |
| PsycINFO | TIAB (Document title and abstract) |
| Cochrane | Title, Abstract, Keyword |
| CINAHL | AB (Abstract) |

**Supplementary material D**. Quality assessment for case series studies (n=14).

| **Criteria** | Bogucki, 2022^28^ | Hu, 2020^33^ | Huang, 2020^34^ | Khawam, 2020^35^ | Edet, 2022^32^ | Naskar, 2022^36^ | Alkhamees, 2021^27^ | Chen, 2020^30^ | Sadeghi, 2021^38^ | Situmorang, 2021^39^ | Callus, 2022^29^ | Nuertey, 2022^37^ | Taube, 2023^40^ | Dinapoli, 2022^31^ |
| --- | --- | --- | --- | --- | --- | --- | --- | --- | --- | --- | --- | --- | --- | --- |
| 1. Was the study question or objective clearly stated? | Yes | Yes | Yes | Yes | Yes | Yes | Yes | Yes | Yes | No | Yes | Yes | No | Yes |
| 2. Was the study population clearly and fully described, including a case definition? | Yes | Yes | Yes | Yes | Yes | Yes | Yes | Yes | Yes | Yes | Yes | Yes | Yes | No |
| 3. Were the cases consecutive? | NA | NA | NA | NA | NA | NA | NA | NA | No | NA | NA | No | NA | No |
| 4. Were the subjects comparable? | NA | NA | NA | NA | NA | NA | NA | NA | CD | NA | NA | No | NA | Yes |
| 5. Was the intervention clearly described? | Yes | Yes | Yes | No | No | No | No | No | No | Yes | Yes | No | No | Yes |
| 6. Were the outcome measures clearly defined, valid, reliable, and implemented consistently across all study participants? | Yes | Yes | Yes | NR | NR | NR | NR | NR | Yes | No | Yes | No | Yes | Yes |
| 7. Was the length of follow-up adequate? | No | No | No | No | No | No | No | No | Yes | No | No | No | No | No |
| 8. Were the statistical methods well-described? | No | No | No | No | No | No | No | No | Yes | No | No | No | No | Yes |
| 9. Were the results well-described? | Yes | No | No | No | No | No | No | No | Yes | No | Yes | No | No | Yes |
| **Total score** | 5/7 | 4/7 | 4/7 | 2/7 | 2/7 | 2/7 | 2/7 | 2/7 | 6/9 | 2/7 | 5/7 | 2/9 | 2/7 | 6/9 |

Note: NA: Not applicable; NR: Not reported; CD: Cannot determine.

**Supplementary material E**. Quality assessment for before-after (pre-post) studies with no control group (n=9)

| **Criteria** | Brennstuhi, 2022^42^ | Compagno, 2022^44^ | Kim, 2020^49^ | Maresca, 2022^57^ | Priyamvada, 2021^59^ | Sun, 2021^63^ | Yang, 2020)^67^ | Biagianti, 2023^41^ | Won, 2022^65^ |
| --- | --- | --- | --- | --- | --- | --- | --- | --- | --- |
| 1. Was the study question or objective clearly stated? | Yes | Yes | Yes | Yes | Yes | Yes | Yes | Yes | Yes |
| 2. Were eligibility/ selection criteria for the study population prespecified and clearly described? | Yes | Yes | No | Yes | Yes | No | No | Yes | Yes |
| 3. Were the participants in the study representative of those who would be eligible for the test/ service/ intervention in the general or clinical population of interest? | Yes | Yes | Yes | Yes | Yes | Yes | Yes | Yes | Yes |
| 4. Were all eligible participants that met the prespecified entry criteria enrolled? | CD | CD | No | CD | CD | NO | CD | No | No |
| 5. Was the sample size sufficiently large to provide confidence in the findings? | No | No | No | Yes | No | No | NR | CR | CD |
| 6. Was the test/service/intervention clearly described and delivered consistently across the study population? | No | No | No | No | Yes | No | No | Yes | No |
| 7. Were the outcome measures prespecified, clearly defined, valid, reliable, and assessed consistently across all study participants? | Yes | Yes | Yes | Yes | Yes | Yes | Yes | Yes | Yes |
| 8. Were the people assessing the outcomes blinded to the participants' exposures/interventions? | No | NR | NR | CD | NR | NR | NR | NR | NR |
| 9. Was the loss to follow-up after baseline 20% or less? Were those lost to follow-up accounted for in the analysis? | CD | CD | Yes | CD | CD | NR | Yes | Yes | Yes |
| 10. Did the statistical methods examine changes in outcome measures from before to after the intervention? Were statistical tests done that provided p values for the pre-to-post changes? | Yes | Yes | Yes | Yes | Yes | Yes | Yes | Yes | Yes |
| 11. Were outcome measures of interest taken multiple times before the intervention and multiple times after the intervention (i.e., did they use an interrupted time-series design)? | Yes | No | Yes | No | No | No | No | No | No |
| 12. If the intervention was conducted at a group level (e.g., a whole hospital, a community, etc.) did the statistical analysis take into account the use of individual-level data to determine effects at the group level? | NA | NA | NA | NA | NA | NA | NA | NA | NA |
| **Total score** | 6/11 | 5/11 | 6/11 | 6/11 | 6/11 | 4/11 | 5/11 | 7/11 | 6/11 |

Note: NA: Not applicable; NR: Not reported; CD: Cannot determine.

**Supplementary material F**. Quality assessment for Controlled Intervention Studies (n=15).

| **Criteria** | Cengiz, 2021^43^ | Fan, 2021^45^ | Kong, 2020^50^ | Li, 2020^52^ | Liu K, 2020^53^ | Liu Y, 2021^54^ | Liu Z, 2021^55^ | Mahendru, 2021^56^ | Parizad, 2021^58^ | Sotoudeh, 2020^62^ | Wei, 2020^64^ | Zheng, 2022^69^ | Rutkowski, 2022^60^ | Shaygan, 2023^61^ | Torbati, 2022^47^ |
| --- | --- | --- | --- | --- | --- | --- | --- | --- | --- | --- | --- | --- | --- | --- | --- |
| 1. Was the study described as randomized, a randomized trial, a randomized clinical trial, or an RCT? | Yes | Yes | Yes | Yes | Yes | No | Yes | Yes | Yes | Yes | Yes | No | Yes | Yes | Yes |
| 2. Was the method of randomization adequate (i.e., use of randomly generated assignment)? | Yes | Yes | Yes | Yes | CD | Yes | Yes | CD | Yes | Yes | Yes | Yes | Yes | Yes | CD |
| 3. Was the treatment allocation concealed (so that assignments could not be predicted)? | Yes | Yes | No | Yes | CD | CD | Yes | CD | Yes | CD | No | CD | Yes | Yes | NR |
| 4. Were study participants and providers blinded to treatment group assignment? | No | No | No | CD | CD | CD | No | CD | No | CD | No | CD | NR | No | NR |
| 5. Were the people assessing the outcomes blinded to the participants' group assignments? | Yes | No | Yes | CD | CD | CD | No | NR | Yes | CD | No | CD | NR | Yes | NR |
| 6. Were the groups similar at baseline on important characteristics that could affect outcomes (e.g., demographics, risk factors, co-morbid conditions)? | No | Yes | Yes | Yes | Yes | Yes | Yes | Yes | Yes | Yes | Yes | No | Yes | Yes | Yes |
| 7. Was the overall drop-out rate from the study at endpoint 20% or lower of the number allocated to treatment? | Yes | Yes | Yes | Yes | Yes | Yes | No | Yes | Yes | No | NR | Yes | NR | Yes | Yes |
| 8. Was the differential drop-out rate (between treatment groups) at endpoint 15 percentage points or lower? | Yes | Yes | Yes | Yes | Yes | Yes | Yes | Yes | Yes | Yes | Yes | Yes | NR | Yes | Yes |
| 9. Was there high adherence to the intervention protocol for each treatment group? | CD | CD | CD | CD | CD | CD | CD | CD | CD | CD | CD | CD | CD | CD | CD |
| 10. Were other interventions avoided or similar in the groups (e.g., similar background treatments)? | Yes | Yes | Yes | Yes | CD | CD | Yes | CD | Yes | Yes | Yes | Yes | Yes | Yes | Yes |
| 11. Were outcomes assessed using valid and reliable measures, implemented consistently across all study participants? | Yes | Yes | Yes | Yes | Yes | Yes | Yes | Yes | Yes | Yes | Yes | Yes | Yes | Yes | Yes |
| 12. Did the authors report that the sample size was sufficiently large to be able to detect a difference in the main outcome between groups with at least 80% power? | Yes | No | NR | Yes | No | Yes | Yes | Yes | Yes | No | No | No | Yes | Yes | NR |
| 13. Were outcomes reported or subgroups analyzed prespecified (i.e., identified before analyses were conducted)? | Yes | No | No | No | No | No | Yes | No | Yes | No | No | No | No | No | No |
| 14. Were all randomized participants analyzed in the group to which they were originally assigned, i.e., did they use an intention-to-treat analysis? | No | Yes | Yes | No | No | Yes | Yes | Yes | Yes | No | Yes | Yes | NR | Yes | Yes |
| **Total score** | 10/14 | 9/14 | 9/14 | 9/14 | 5/14 | 7/14 | 10/14 | 7/14 | 12/14 | 6/14 | 7/14 | 6/14 | 7/14 | 11/14 | 7/14 |

Note: NA: Not applicable; NR: Not reported; CD: Cannot determine.

**Supplementary material G**. Non-randomized controlled studies (n=5)

| **Criteria** | Ghodrati-Torbati, 2022 ^48^ | Yuan, 2021^68^ | Xiao, 2020^66^ | Ganesan, 2023^46^ | Lerthattasilp, 2021^51^ |
| --- | --- | --- | --- | --- | --- |
| 1. Was the study described as randomized, a randomized trial, a randomized clinical trial, or an RCT? | No | No | No | No | No |
| 2. Was the method of randomization adequate (i.e., use of randomly generated assignment)? | Yes | NR | No | No | No |
| 3. Was the treatment allocation concealed (so that assignments could not be predicted)? | Yes | NR | No | No | No |
| 4. Were study participants and providers blinded to treatment group assignment? | CD | NR | NR | No | NR |
| 5. Were the people assessing the outcomes blinded to the participants' group assignments? | CD | NR | NR | No | NR |
| 6. Were the groups similar at baseline on important characteristics that could affect outcomes (e.g., demographics, risk factors, co-morbid conditions)? | Yes | Yes | Yes | Yes | No |
| 7. Was the overall drop-out rate from the study at endpoint 20% or lower of the number allocated to treatment? | Yes | Yes | Yes | Yes | Yes |
| 8. Was the differential drop-out rate (between treatment groups) at endpoint 15 percentage points or lower? | Yes | Yes | Yes | Yes | Yes |
| 9. Was there high adherence to the intervention protocols for each treatment group? | CD | CD | CD | CD | CD |
| 10. Were other interventions avoided or similar in the groups (e.g., similar background treatments)? | NR | Yes | Yes | Yes | Yes |
| 11. Were outcomes assessed using valid and reliable measures, implemented consistently across all study participants? | Yes | Yes | Yes | Yes | Yes |
| 12. Did the authors report that the sample size was sufficiently large to be able to detect a difference in the main outcome between groups with at least 80% power? | Yes | NR | No | Yes | No |
| 13. Were outcomes reported or subgroups analyzed prespecified (i.e., identified before analyses were conducted)? | No | No | No | No | No |
| 14. Were all randomized participants analyzed in the group to which they were originally assigned, i.e., did they use an intention-to-treat analysis? | Yes | Yes | Yes | No | No |
| **Total score** | 8/14 | 6/14 | 6/14 | 6/14 | 4/14 |

Note: NA: Not applicable; NR: Not reported; CD: Cannot determine
